# Identification of novel small molecule inhibitors of twin arginine translocation (Tat) pathway and their effect on the control of *Campylobacter jejuni* in chickens

**DOI:** 10.3389/fmicb.2024.1342573

**Published:** 2024-04-17

**Authors:** Loïc Deblais, Mary Drozd, Anand Kumar, Janet Antwi, James Fuchs, Rahul Khupse, Yosra A. Helmy, Gireesh Rajashekara

**Affiliations:** ^1^Department of Animal Sciences, The Ohio State University, OARDC, Wooster, OH, United States; ^2^School of Veterinary Medicine and Biomedical Sciences, University of Nebraska-Lincoln, Lincoln, NE, United States; ^3^Los Alamos National Laboratory, Bioscience Division, Group B-10: Biosecurity and Public Health, Los Alamos, NM, United States; ^4^Division of Medicinal Chemistry and Pharmacognosy, College of Pharmacy, The Ohio State University, Columbus, OH, United States; ^5^College of Pharmacy, University of Findlay, OH, United States

**Keywords:** *Campylobacter jejuni*, poultry production system, twin arginine translocase, small molecule inhibitor, microbiome

## Abstract

**Introduction:**

Control of *Campylobacter* from farm to fork is challenging due to the frequent emergence of antimicrobial-resistant isolates. Furthermore, poultry production systems are known reservoirs of *Campylobacter*. The twin-arginine translocation (Tat) pathway is a crucial bacterial secretion system that allows *Campylobacter* to colonize the host intestinal tract by using formate as the main source of energy. However, Tat pathway is also a major contributing factor for resistance to copper sulfate (CuSO_4_).

**Methods:**

Since mammals and chickens do not have proteins or receptors that are homologous to bacterial Tat proteins, identification of small molecule (SM) inhibitors targeting the Tat system would allow the development of safe and effective control methods to mitigate *Campylobacter* in infected or colonized hosts in both pre-harvest and post-harvest. In this study, we screened 11 commercial libraries (*n* = 50,917 SM) for increased susceptibility to CuSO_4_ (1 mM) in *C. jejuni* 81–176, a human isolate which is widely studied.

**Results:**

Furthermore, we evaluated 177 SM hits (2.5 μg/mL and above) that increased the susceptibility to CuSO_4_ for the inhibition of formate dehydrogenase (Fdh) activity, a Tat-dependent substrate. Eight Tat-dependent inhibitors (T1–T8) were selected for further studies. These selected eight Tat inhibitors cleared all tested *Campylobacter* strains (*n* = 12) at >10 ng/mL in the presence of 0.5 mM CuSO_4_*in vitro*. These selected SMs were non-toxic to colon epithelial (Caco-2) cells when treated with 50 μg/mL for 24 h and completely cleared intracellular *C. jejuni* cells when treated with 0.63 μg/mL of SM for 24 h in the presence of 0.5 mM of CuSO_4_. Furthermore, 3 and 5-week-old chicks treated with SM candidates for 5 days had significantly decreased cecal colonization (up to 1.2 log; *p* < 0.01) with minimal disruption of microbiota. *In silico* analyses predicted that T7 has better drug-like properties than T2 inhibitor and might target a key amino acid residue (glutamine 165), which is located in the hydrophobic core of TatC protein.

**Discussion:**

Thus, we have identified novel SM inhibitors of the Tat pathway, which represent a potential strategy to control *C. jejuni* spread on farms.

## Introduction

*Campylobacter* is a leading cause of bacterial foodborne gastroenteritis worldwide (Igwaran and Okoh, 2019) and a major public health problem. A recent estimate by the CDC indicates that *Campylobacter* is not only among the most common causes of domestically acquired foodborne illnesses in humans (over 845,024 cases per year) but also is among the leading causes of hospitalization (over 8,463 annually) in the United States (USDA ERS–Cost Estimates of Foodborne Illnesses, 2017; Igwaran and Okoh, 2019).

Poultry products represent a key source of human *Campylobacter* infections (Kittl et al., 2013; Skarp et al., 2016). *C. jejuni* and *C. coli* densely colonize the intestine of poultry (up to 10^9^ bacteria per gram of ceca in chicken) and are the most common species encountered in human infections (Allos, 2001; Newell and Fearnley, 2003; Lee and Newell, 2006; Young et al., 2007; Facciolà et al., 2017; Connerton et al., 2018). Despite extensive intestinal colonization, *Campylobacter* infection produces little or no clinical diseases in poultry (Johnson et al., 2017). Prevalence studies conducted in Europe and United States have reported *Campylobacter*-positive flocks ranging up to 100% (Newell and Fearnley, 2003; Luangtongkum et al., 2006; Jorgensen et al., 2011; Sibanda et al., 2018; Koutsoumanis et al., 2020; Kuhn et al., 2020). Colonized chickens shed *Campylobacter* in their feces until slaughter (up to 10^9^ CFU/g of cecum) (Newell and Fearnley, 2003; Luangtongkum et al., 2006; Jorgensen et al., 2011; Sibanda et al., 2018; Koutsoumanis et al., 2020; Kuhn et al., 2020), increasing the risk of horizontal transmission of *Campylobacter* to the whole flock and post-harvest contaminations of the meat products (over 50% in average and up to 100%) (Logue et al., 2003; Berrang et al., 2007; Young et al., 2007; Guerin et al., 2010; Hue et al., 2010; Kaakoush et al., 2015). The high prevalence of *Campylobacter* in poultry and detection of identical genotypes in both poultry and human isolates support poultry contamination as the predominant route of human infection (Allos, 2001; Newell and Fearnley, 2003; Lee and Newell, 2006; Young et al., 2007; Facciolà et al., 2017; Connerton et al., 2018).

On-farm *Campylobacter* control efforts have not been successful in mitigating human *Campylobacter* infections as evidenced by the continuous increase in human infections (Kittl et al., 2013; Skarp et al., 2016). Although improving biosecurity has beneficial effects on lowering the overall flock prevalence (Kaakoush et al., 2015), these measures have not resulted in consistent and predictable outcomes in controlling *Campylobacter* (Wagenaar et al., 2013; Kaakoush et al., 2015). In addition, stringent biosecurity measures are cost-prohibitive and hard to maintain and their effectiveness seems to vary with production systems and they do not apply to small farm poultry operations (e.g., backyard chickens) and pet chickens (Koutsoumanis et al., 2020). Currently, there are no effective competitive exclusion products, vaccines, bacteriocins, bacteriophages, or feed/water additives to exclude *Campylobacter* from chickens under production conditions (Lin, 2009; Richards et al., 2019; Vandeputte et al., 2019; Quintel et al., 2020; Hakeem and Lu, 2021). Furthermore, the widespread use of antibiotics in poultry production has been implicated in the emergence of highly resistant *Campylobacter* strains (Young et al., 2007; Agyare et al., 2018; Yang et al., 2019; Andrew Selaledi et al., 2020). Therefore, there is a critical need for novel anti-*Campylobacter* control strategies that can amend and/or replace on-going efforts and specifically target pathways and novel mechanisms to combat both *Campylobacter* spread and antibiotic resistance.

The transport of proteins from cytoplasm to extra-cytoplasmic locations is critical for bacterial survival, virulence, and stress resistance (Berks, 2015). Extra-cytoplasmic protein transport in bacteria most commonly occurs via two major export systems, namely, the general secretory (Sec) pathway and the twin-arginine translocation (Tat) pathway (Frain et al., 2019a,b). The *Campylobacter* Tat pathway is highly conserved between strains, and the inactivation of the Tat pathway induces a multitude of functional defects in *Campylobacter* including motility, outer membrane permeability, biofilm formation, antibiotic resistance, growth under oxygen-limiting conditions, iron acquisition, and copper homeostasis (Drozd et al., 2011; Kassem et al., 2011). Copper is crucial for the survival of pathogenic bacteria in the host and external environment; however, it also exhibits antimicrobial properties at high concentration (Hall et al., 2008; Stolle et al., 2016). As a defense mechanism, *C. jejuni* expresses two important proteins for copper homeostasis as follows: (1) CopA a copper transporting P-type ATPase responsible for transporting toxic Cu (I) from cytoplasm to periplasm, (2) CueO, a multicopper oxidase that converts toxic Cu (I) into the less toxic Cu (II) in periplasm and is dependent on the Tat system. Both of these copper homeostasis proteins are essential for copper resistance and avian host colonization (Hall et al., 2008; Stolle et al., 2016; Kassem et al., 2017; Gardner and Olson, 2018). In addition, the deletion of the *tatC* gene significantly reduced *C. jejuni* persistence in the intestinal tract of chickens and decreased fecal shedding (Rajashekara et al., 2009). Previous studies have demonstrated that Tat inhibitors are an effective control method to mitigate *Pseudomonas aeruginosa* (Vasil et al., 2012; Massai et al., 2019) and *Escherichia coli* (Panahandeh et al., 2008; Bageshwar et al., 2016; Blümmel et al., 2017). Furthermore, the use of SM has been shown to be effective strategy against multi-drug resistant pathogens (i.e., *Salmonella*, *Escherichia coli*, *Campylobacter*, *Staphylococcus*, *Burkholderia*, *Pseudomonas*, and *Candida*), where conventional antibiotics failed (Hong-Geller and Micheva-Viteva, 2013; Abouelhassan et al., 2014; Selin et al., 2015; Guo et al., 2016; Deblais et al., 2018, 2019; Helmy et al., 2018, 2020; Kathayat et al., 2018). Since chickens and mammals do not have Tat protein homologs, the inactivation of the Tat pathway using small molecule (SM) inhibitors is a promising approach to discover new antimicrobial agents with no toxicity to eukaryotes as an alternative to conventional *Campylobacter* control methods.

In this study, we used commercially available SM libraries (n = 50,917) to identify several, lead SM inhibitors that targeted the Tat-system and had anti-*C. jejuni* activity. Tat-dependent inhibitors were identified by (1) increased *C. jejuni* susceptibility to copper sulfate and (2) reduced *C. jejuni* Fdh activity. Eight potential Tat-dependent inhibitors (T1-T8) exhibited *in vitro* antimicrobial activity against *C. jejuni*. Two inhibitors (T2 and T7) were identified as promising lead compounds with good antimicrobial efficacy against *C. jejuni* in chickens and with minimal impact on the host microbiota. Furthermore, an *in silico* docking study identified that the most promising Tat inhibitor (T7) targets key amino acids in TatC.

## Materials and methods

### Bacterial strains

*Campylobacter jejuni* 81–176 was the primary model strain used for the selection of the potential Tat-dependent inhibitors by high-throughput screening. *C. jejuni* 81–176 is resistant to 0.5 and 1 mM CuSO_4_ (Supplementary Figure S1). *C. jejuni* 81–176 *tatC* knockout mutant (*C. jejuni ΔtatC*) was used to confirm the Tat-dependent inhibitory effect of the selected SM. *C. jejuni ΔtatC* is susceptible to 0.5 mM and higher of CuSO_4_ (Rajashekara et al., 2009). The specificity of the Tat-dependent inhibitors was also tested on 11 additional *Campylobacter* strains (*C. coli* ATCC33559 and 10 other *C. jejuni* strains isolated from chickens) and 7 commensal/beneficial bacteria (Supplementary Table S1). The 1 *C. jejuni* strains (Au-13, Au-20, Au-38, Au-39, Au-47, Au-50, Au-24, Au-32, Au-44, and Au-45) were selected based on their single nucleotide polymorphism (SNP)-type and percent of prevalence in chickens (Merchant-Patel et al., 2008; Kumar et al., 2016). Additional details about the bacterial strains are shown in Supplementary Table S1.

### Eukaryotic models

Colonic epithelial cells (Caco-2) were used to evaluate the cytotoxicity and the ability of the selected eight SMs to clear intracellular *C. jejuni*. The Caco-2 cells were obtained from the American Type Culture Collection (ATCC HTB-37; Rockville, MD, United States). For *in-vivo* testing, 3 and 5-week-old White Cronish broiler chickens, obtained from Meyer’s hatchery (Polk, OH, USA), were used to validate the anti-*C. jejuni* efficacy (log reduction in cecal content) of the selected four SMs (T1, T2, T7, and T8) in poultry and their impact on the cecal microbiota. Broiler chickens were grown in accordance with The Ohio State University Animal Care and Use Program (accredited by the Association for Assessment and Accreditation of Laboratory Animal Care International) and performed following the Institutional Animal Care and Use Committee. Chickens were fed a standard broiler diet (i.e., broiler starter phase from day 0 to 10; broiler growth phase from day 11 to 25; and broiler finisher phase from day 26 to 42; PNW extension #658) which was obtained from local feed mill at the Ohio Agricultural Research and Development Center (OARDC). Chickens and associated rooms were observed at least twice daily to assure that no shortage in feed or side effects occur due to the bacterial inoculation or treatments provided during the experiments.

### SM libraries

A total of 50,917 SMs obtained from 11 libraries were screened in this study. SMs were suspended in 100% DMSO at concentration ranging from 2.5 μg/mL to 12.5 μg/mL between libraries and stored at −80°C (Supplementary Table S2). These libraries originated from The National Screening Laboratory for the Regional Center of Excellence in Biodefense and Emerging Infectious Disease (NSRB, United States)—The New England Regional Center of Excellence for Biodefense and Emerging Infectious Diseases (NERC, USA) collection. The NSRB and NERC libraries included FDA-approved bio-active SM, SM used by NIH in recent clinical trials, bio-active screens, and diversified SM synthesized for favorable physico-chemical properties (e.g., solubility, low/no toxicity and increased stability). Details about the origin and concentration of the libraries are shown in Supplementary Table S2.

### High-throughput copper sulfate sensitivity assay

Our *in silico* study highlighted that the unique assembly and disassembly feature of Tat system is required for the translocation of several relatively large folded proteins, such as formate dehydrogenase (Fdh) and multi-copper oxidase (CueO) (Rajashekara et al., 2009). Since CueO function requires the transport of the Tat system, increased susceptibility to copper was used as an indicator during our *in vitro* screen for Tat-specific SM inhibitors. The identification of SM increasing sensitivity of *C. jejuni* 81–176 to copper sulfate (1 mM CuSO_4_, non-lethal dose) was performed using a high-throughput screening in 384 plate formats at NSRB facilities (Harvard Medical School, Cambridge, MA, USA). Columns 1 to 24 of assay plates were filled with 40 μL of MH broth +1 mM CuSO_4_ using a Matrix WellMate (Thermo Fisher, United States) automatic plate filler (Drozd, 2012). In total, 100 nL of SM were transferred to each well using The Institute of Chemistry and Cell Biology (ICCB, USA) Longwood Screen Facility Seiko D-TRAN XM3106-31 PN 4-axis cartesian robot (V&P Scientific), which was controlled by SRC-310A Controller/SPEL. Columns 1–23 of the assay plates were inoculated with 40 μL of *C. jejuni* 81–176 normalized at optical density (OD_600_) of 0.16 in fresh MH broth supplemented with 1 mM CuSO_4_. Wells in column 24 were filled with 40 μL of *C. jejuni ΔtatC* (susceptible to 1 mM CuSO_4_) normalized at 0.16 OD_600_ in the same conditions. The *ΔtatC* mutant is susceptible to 0.5 mM CuSO_4_; however, for screening purpose, we used the stringent 1 mM CuSO_4_. Plates were incubated at 42°C for 36 h under microaerophilic conditions (85% N_2_, 10% CO_2_, and 5% O_2_). Turbidimetric measurements (OD_600_) were recorded before and after incubation using a Biotek Synergy HT spectrophotometer. The positive and negative controls on each assay plate were used to calculate a Z’ value for that plate. If the Z’ value was >0, the threshold for defining a compound as positive was set at three standard deviations above the average positive control value. Z’ is calculated as described in the study mentioned in the reference (Zhang et al., 1999): Z’ = 1- (3σneg + 3σpos)/(μneg-μpos), where μ is the mean and σ is the standard deviation. The Δ*tatC* mutant is the positive control mimic, and wild type without drug is the negative control.

Furthermore, validation of the primary assay was performed in a 96-well plate format for the selected 177 SMs with high drug-like properties that significantly increased susceptibility of *C. jejuni* to CuSO_4_. In total, 100 μL of *C. jejuni* suspension normalized at 0.08 OD_600_ in fresh MH broth supplemented with 1 mM CuSO_4_ were transferred to each well of a 96-well plate and treated with 6.25 μg of SM. A parallel 96-well plate was prepared as described above but without CuSO_4_, to identify SM that did not inhibit the growth of *C. jejuni* in a Tat-specific manner. Plates were incubated at 42°C under microaerophilic conditions for 24 h. Turbidimetric measurements were recorded before and after incubation, to remove a potential increase in OD_600_ caused by the SM. Only SM inhibiting *C. jejuni* WT growth in the presence of CuSO_4_ without inhibiting *C. jejuni* WT growth in the absence of CuSO_4_ was selected for further analyses.

### Counter screens using the screensaver SM database

Based on previous publicly available bioassay data, the ICCB-longwood/NSRB Screensaver database version v2010.10.29 and v2012.01.26 was used to eliminate SMs that were less likely to target the Tat pathway (Tolopko et al., 2010). Commercial and pharmaceutical based libraries with known antibacterial applications were cross-referenced with the selected SM. In addition, SMs that had positive effects on eukaryote-based screens were deprioritized because of the absence of eukaryotic homolog Tat system.

### Counter-screen using medicinal chemistry software

A series of filters were established to select SM with the optimized drug-like properties. The criteria were based on physicochemical descriptors, potential liabilities (i.e., predicted toxicity), chemical structural diversity, and novelty. ChemDraw suite (CambridgeSoft, PerkinElmer, United States) was used to calculate molecular weight from the simplified molecular-input line-entry system (SMILES) notation. Molecular weights less than 200 Daltons (Da) and more than 550 Da were deprioritized due to the golden triangle measurements for drug-like characteristics (Johnson et al., 2009). ChemBioFinder (CambridgeSoft, PerkinElmer, United States) was used to identify structural redundancy between the SM and remove highly similar candidates. SciFinder® (Chemical Abstracts Service) was used to deprioritize SM, showing more than 90% similarity with previously investigated drugs.

### Formate dehydrogenase inhibition activity assay

Formate is an essential source of energy for *Campylobacter* and plays a role in optimizing the adaptation of *C. jejuni* to the oxygen-limited gastrointestinal tract of the host (Kassem et al., 2017). Fdh is translocated by the Tat system in the periplasm, and thus, the inactivation of Tat system results in a reduction of the Fdh activity. Hence, this activity was used as an indicator during our *in vitro* screening procedures, to validate Tat-dependent SM inhibitors of *C. jejuni*. The Fdh inhibition activity assay was performed with the 177 SM with drug-like properties that increased the susceptibility of *C. jejuni* to CuSO_4_ (1 mM). *C. jejuni* 81–176 suspension normalized to 0.08 OD_600_ in fresh MH broth was incubated for 24 h in microaerophilic condition with 6.25 μg of SM. Subsequently, the treated cultures were suspended in an oxygen-restricted solution containing 25 mM sodium phosphate buffer (pH 7) with 1 mM benzyl-viologen and 10 mM sodium formate. The increase of OD_578_ was measured using a SpectraMax Plus 384 absorbance plate reader (Molecular Devices, USA), to monitor the reduction in benzyl viologen, an indicator of Fdh activity. *N* = three replicates per SM. *C. jejuni* 81–176 treated with 1% DMSO and *C. jejuni* ∆*tatC*mutant were used as controls. SMs that inhibited at least 30% Fdh activity (which is equivalent to the inhibitory effect of 1 mM of azide on FDH activity (Davies, 2017)) compared with the DMSO control were further down selected for analysis.

### Activity spectrum of the selected 19 SMs against several *Campylobacter jejuni*, *Campylobacter coli*, and commensal/beneficial gut bacteria

In total, 19 SMs were selected using CuSO_4_ sensitivity and Fdh inhibition assay and further tested on 11 *Campylobacter* strains grown in MH broth supplemented with 0.5 mM CuSO4 and treated with 6.25 μg/mL of SM, as described above. A similar experiment was performed with seven commensal/beneficial gut bacteria to determine species specificity of these SMs. In brief, an overnight suspension was normalized at 0.05 OD_600_ using the appropriate medium and challenged with 6.25 μg/mL of SM. Details about the strains and their growing conditions are shown in Supplementary Table S1. Medium alone and 1% DMSO were used as controls (*N* = 3 replicates per SM).

### Copper sulfate sensitivity dose–response assay *in vitro* using the selected 19 SM

A CuSO_4_ sensitivity dose–response assay was performed to determine the minimal concentration of SM that completely inhibited (MIC) or killed (MBC) *C. jejuni* 81–176 in the presence of CuSO_4_ (0.5 mM). In total, 19 SMs were two-fold serially diluted to obtain a final SM concentration ranging from 6.25 to 0.012 μg/mL. *C. jejuni* 81–176 was then treated with a determined concentration of SM, as described in the copper sensitivity assay. The lowest SM concentration that completely inhibited the growth without killing *C. jejuni* in the presence of 0.5 mM CuSO_4_ was considered the MIC (no increase in OD_600_ over time, but viable cells were recovered on agar plate after challenge). The lowest bactericidal SM concentration was considered the MBC (no increase in OD_600_ over time and no viable cells were recovered on agar plate after challenge) (*N* = 3 replicates per SM). A similar copper sulfate sensitivity dose–response assay was performed with the eight most potent SMs (T1-T8) against other *Campylobacter* strains (*n* = 11; Supplementary Table S1) using methodology described above. However, these SMs were two-fold serially diluted to obtain a final SM concentration ranging from 5 μg to 0.0625 μg for testing (*N* = two independent experiments with four technical replicate for each SM).

### Cytotoxicity of the selected eight SM on Caco-2 colon epithelial cells

Cytotoxicity of the eight SMs (T1-T8) was tested on Caco-2 cells at 5, 25, and 50 μg/mL, as previously described (Kumar et al., 2016; Deblais et al., 2019). In brief, a 96-well plate seeded with approximately 1.4 × 10^5^ Caco-2 cells/well in MEM medium was challenged with a final SM concentration of 5, 25, and 50 μg/mL. After 24 h of incubation at 37°C in a humidified 5% CO_2_ incubator, cytotoxic effects were determined using the PierceTM Lactate Dehydrogenase Cytotoxicity Assay Kit (Thermo Fisher Scientific). Cell death was measured based on the production of formazan (chromogenic dye), which can be read at OD_570_. Equal concentrations (5, 25, and 50 μg/mL) of kanamycin or chloramphenicol, 1% DMSO, and 10X lysis buffer were used as control. The cytotoxicity level was calculated according to the manufacturer’s instructions (*N* = two independent experiments with three technical replicate for each SM).

### Copper sulfate sensitivity dose–response assay in infected Caco-2 colon epithelial cells

The intracellular reduction in *C. jejuni* in the presence of a given SM (T1-T8) and 0.5 mM CuSO_4_ were evaluated using Caco-2 cells, as previously described (Kumar et al., 2016; Deblais et al., 2019). In brief, cells were infected for 2 h using a multiplicity of infection (MOI) of 100. Infected cells were treated with SM at a final concentration ranging between 5 and 0.315 μg/mL and incubated at 37°C for 24 h in a humidified 5% CO_2_ incubator. Following incubation, cells were washed once with 1X PBS, lysed with 0.1% Triton-100X, serially 10-fold diluted in 1X PBS, and plated on MH agar plate. Plates were incubated in microaerophilic conditions at 42°C for 24 h, to determine the intracellular survival. Cells not infected and not treated and cells infected and treated with 1% DMSO were used as controls (*N* = two independent experiments with four technical replicate for each SM).

### Effect of selected four SMs on *Campylobacter jejuni* persistence in chicken ceca

Four SMs (T1, T2, T7, and T8) were selected from the initial list of eight SMs based on their low MBC in Caco2 cells (<5 μg SM/ml), low cytotoxicity indexes (<20% toxicity at 5 μg SM /ml), and their minimal effect on commensal bacterial species (less than two species inhibited by the SM out of the seven strains tested; Table 1). The antimicrobial efficacy of our SM was tested on 5-week-old chickens, to assess the clearance of *C. jejuni* load right before slaughter age. Overall, 5-week-old *Campylobacter*-free, i.e., specific pathogen-free [SPF] chickens were inoculated orally with a mixture of five *C. jejuni* strains (10^5^ CFU/chicken, Supplementary Table S3). Rectal swabs were collected 1 day post-inoculation (DPI) to confirm *C. jejuni* colonization (CFU/g of feces) in birds. From 2 DPI to 7 DPI, groups of 4–5 chickens were treated orally twice a day with one of the four SMs (T1, T2, T7, or T8; approximately 0.225 mg of SM per kg body weight). Details of the treatment groups and the inoculum are shown in Supplementary Table S3.

**Table 1:** Antimicrobial properties of the eight most potent Tat-dependent inhibitors.

SM	*C. jejuni* 81–176 *in vitro*		Growth inhibition at 6.25 μg/mL		*C. jejuni* 81–176 MBC in Caco2 cells (μg /ml)
	MIC (μg/ml)	MBC (μg/ml)	*Campylobacter* spp.	Commensal/beneficial gut bacteria	
T1	0.19	0.62	12/12	0/7	0.63
T2	0.01	0.62	12/12	2/7 (Lb and Ef)	1.25
T3	0.04	>5	12/12	2/7 (LGG and Ef)	5
T4	0.01	>5	12/12	1/7 (Ef)	10
T5	0.01	0.04	12/12	1/7 (Ef)	10
T6	1.25	2.5	12/12	0/7	10
T7	0.02	0.16	12/12	1/7 (LGG)	2.5
T8	0.08	0.12	12/12	2/7 (Lb and Ef)	5

MIC: minimal bacteriostatic concentration (μg/ml) in presence of 0.5 mM CuSO_4_; MBC: minimal bactericidal concentration (μg/ml) in presence of 0.5 mM CuSO_4_; >5: the MBC was higher than 5 μg/mL. Ef, *Enterococcus faecalis*; Lb, *Levilactobacillus brevis*; LGG, *Lacticaseibacillus rhamnosus* GG. Details about the *Campylobacter* and commensal/beneficial strains are displayed in Supplementary Table S1.

The antimicrobial efficacy of T1, T7, and T8 was also tested on 3-week-old chickens to assess the clearance of *C. jejuni* load in chicken right after inoculation. Overall, 3-week-old *Campylobacter*-free chickens were inoculated orally with a mixture of five *C. jejuni* strains (10^5^ CFU/chicken, Supplementary Table S3). Rectal swabs were collected 1 DPI to confirm the intestinal colonization of the birds by *C. jejuni* (CFU/g of feces). From 2 DPI, chickens were treated orally twice a day for 5 days (from 2 DPI to 7 DPI) with one of the SMs (T1, T7, or T8; approximately 0.127 mg of SM per kg body weight; n = 5–6 chicken per group). Details of the treatment groups and the inoculum are shown in Supplementary Table S3.

For both experiments, *C. jejuni* colonized-chickens treated with 0.0001% DMSO and colonized chickens without additional carrier treatment (non-treated) were used as controls (*n* = 3–6 chicken per group). Details of the treatment groups and the inoculum are shown in Supplementary Table S3. Both 3 and 5-week-old chickens were euthanized at 7 DPI using carbon dioxide gas and ceca (cecal content and the pouch) and were aseptically collected. One of the ceca pairs was immediately stored at −80°C for microbiota studies. The other ceca were suspended in 1X PBS, homogenized, serially diluted, plated on MH media supplemented with *Campylobacter* Selective Supplement (CSS) agar plate, and incubated for 48 h at 42°C in microaerophilic conditions to determine *C. jejuni* load in the ceca (CFU/g of ceca).

### DNA extraction and 16S MiSeq sequencing

All the samples collected during the chicken experiments (n = 58) were selected for microbiota analysis. Genomic DNA was extracted from 150 to 200 mg of cecal contents using the PureLink Microbiome DNA Purification Kit (Life Technologies, Invitrogen Corp.) and combined with RNAse treatment (10 units/h). After quality control with nanodrop, the 16S rRNA V4-V5 variable region was amplified, purified, and sequenced. Amplicon libraries were prepared by using Phusion® High-Fidelity PCR Kit (New England Biolabs Inc., Ipswich, MA, United States), as previously described (Kumar, 2015; Deblais et al., 2018, 2019; Kumar et al., 2018; Srivastava et al., 2020). PCR products were cleaned using AMPure XP PCR (Beckman Coulter Inc., Beverly MA, USA) and were sequenced using Illumina MiSeq 300-base paired-end kit at the Molecular and Cellular Imaging Center.^1^ Sequencing raw data files are publicly available at NCBI Bioproject #PRJNA1023035.

### Bioinformatics analysis

Quality control of the raw reads was performed using FastQC (Babraham Bioinformatics, Cambridge, United States). Trimmomatic was used for trimming and removal of NexteraPE-PE adapter sequences (Bolger et al., 2014). Trimmed reads were processed using QIIME2 v. 2020.11 (Bolyen et al., 2019). The DADA2 plugin was used to process and check the quality of the reads (Callahan et al., 2016). A sequencing depth of 6,600 reads was used for the rarefaction. Taxonomic assignment was performed using QIIME2 and the latest SILVA reference database (version 138.1; 99% homology cut off) (Quast et al., 2013). The obtained reads were further filtered for eukaryotic, mitochondrial, and chloroplastic genetic signatures.

### *In silico* docking study of the interactions between the selected compounds and the Tat system

Autodock 4.0 (BIOVIA discovery studio visualizer) (Morris et al., 2009) was used for docking with a homology model, which was generated using an online platform Phyere 2 (Kelley et al., 2015). Due to the unavailability of the 3D crystal structure of *C. jejuni* TatC, we turned to *Aquifex aeolicus* VF5, which possessed a TatC homolog. The sequence alignment and secondary structure of *A. aeolicus* VF5 closely resemble those of *C. jejuni* TatC (Ramasamy et al., 2013). Of particular significance, the 3D crystal structure of *A. aeolicus* VF5 is readily accessible in the Protein Data Bank (PDB ID: 4HTS). Consequently, we opted to utilize this structure for the generation of a homology model and Discovery Studio Visualizer along with Chimera to visualize protein–ligand (SM) interaction. Graphical User Interface: AutoDock Tool (ADT) was used for the preparation of pdbqt files for protein and ligand and grid box creation. AutoGrid was employed for the preparation of the grid map, and the grid size was set to 60X60X60 xyz points with grid spacing at 0.375 A. During docking, both protein and ligand were considered as rigid, and the outcomes of docking with 1.0 A in positional root-mean-square deviation (RMSD) were clustered together. The docking pose with most favorable parameters (i.e., lowest energy or binding affinity) was aligned with protein structure and was further analyzed using BIOVIA discovery studio visualizer.

### Data analysis

A one-way ANOVA combined with a Tukey’s test was used to analyze the difference in the abundance of *C. jejuni* in ceca between treatments. A *p*-value ≤0.05 was considered statistically significant. Alpha diversity was analyzed using Shannon (richness and evenness) and Faith’s PD (phylodiversity) indices. The Kruskal–Wallis test was used to identify difference in alpha diversity. Based on Bray–Curtis distance matrices, permutational multivariate analysis of variances (PERMANOVA) was used to identify difference in beta diversity (unweighted and weighted uniFrac). An analysis of composition of microbes (ANCOM) was used to identify differences in the relative abundance between phylum and species levels (Mandal et al., 2015). A *p*-value ≤0.01 was considered statistically significant. A multivariate analysis was performed to identify potential correlations between the spectrum of activity and antimicrobial efficacy (MIC/MBC) of the selected SM and the microbiota data and the *C. jejuni* load in the chicken ceca. Table 2 shows summary of the cutoff selection criteria used in this study, to select the best Tat-dependent SM inhibitors (Ramasamy et al., 2013).

**Table 2:** Selection criteria used in this study to identify the most potent Tat-dependent SM inhibitors.

Experiment	Selection criteria	SM concentration tested	Tool used	Measuring method	Number of SM tested	Number of SM selected
Primary screening – CuSO_4_ sensitivity assay	Select SM that inhibited *C. jejuni* 81–176 growth only in the presence of sub-lethal dose of CuSO_4_ (1 mM)	2.5 to 12.5 μg	384-well plate + *C. jejuni* 81–176	Turbidimetry (cell growth at 600 nm)	50,917	679
Validation of primary screening	Select SM that inhibited *C. jejuni* 81–176 growth only in the presence of sub-lethal dose of CuSO_4_ (1 mM)	2.5 to 12.5 μg	96-well plate + *C. jejuni* 81–176	Turbidimetry (cell growth at 600 nm)	679	679
Counter *in silico* screening	Eliminate less likely to target the Tat pathway	NA	ICCB-longwood/NSRB Screensaver database version	No known activity against eukaryote hosts	679	177
	Select SM with the optimized drug-like properties (MW between 200 and 500 Da)	NA	ChemDraw + Golden triangle measurements	Lipinski rule of 5 MW (Da)		
	Eliminate SM with structural similarities higher than 90%	NA	ChemBioFinder + SciFinder	Structural similarity		
FDH activity inhibition assay	Select SM that inhibited at least 30% of *C. jejuni* 81–176 FDH activity	6.25 μg	384-well plate + *C. jejuni* 81–176	Turbidimetry (reduction of benzyl viologen at 578 nm)	177	21
CuSO_4_ sensitivity dose–response assay *in vitro*	Identify MIC and MBC against *C. jejuni* 81–176 in the presence of sub-lethal dose of CuSO_4_ (0.5 mM)	0.012 to 6.25 μg	96-well plate + *C. jejuni* 81–176	Turbidimetry (cell growth at 600 nm) + cell viability	19	8
Antimicrobial activity of SM on *Campylobacter* strains and commensal /beneficial bacteria	Select SM that had completely inhibited the growth of the 11 other *Campylobacter* strains in the presence of sub-lethal dose of CuSO_4_ (0.5 mM)	6.25 μg	96-well plate + *Campylobacter* isolates (*n* = 11) or commensal/beneficial bacteria (*n* = 7)	Turbidimetry (cell growth at 600 nm) + cell viability		
	Select SM that affected the growth of less than 2/7 commensal/beneficial bacteria in the presence of sub-lethal dose of CuSO_4_ (0.5 mM)					
Cytoxicity of SM on colon epithelial cells	Select SM that displayed less than 20% cytotoxicity on Caco2 colon epithelial cells treated with 5 μg	5 to 50 μg	96-well plate + Caco2 colon epithelial cells	Turbidimetry (production of formazan at 570 nm)	8	4
CuSO_4_ sensitivity dose–response assay in infected colon epithelial cells	Select SM that have MBC below 5 μg against intracellular *C. jejuni* 81–176	0.315 to 5 μg	96-well plate + infected Caco2 colon epithelial cells	cell viability		
Antimicrobial efficacy of the SM on colonized chickens	Select SM that significantly reduce *C. jejuni* load in ceca, while having no impact on cecal microbiota and body weight	0.127 and 0.225 mg of SM per kg body weight for 3- and 5-week-old chickens, respectively	Colonized 3- and 5- week-old broiler chickens	Body weight, *C. jejuni* load in ceca, and cecal microbiota	4	2
Docking study	Identify storng binding affinity of our SM on Tat proteins	NA	Autodock 4.0	Binding affinity	2	2

*All microbiology experiment presented above were conducted by growing *C. jejuni* and *C. coli* in MH broth for 24 h in microaerophilic condition at 42°C. CuSO_4_: copper sulfate; FDH, Formate dehydrogenase; NA, not applicable; SM, small molecule; MW, molecular weight.

## Results

### The growth of *Campylobacter jejuni* 81–176 affected by 177 SMs only in the presence of copper sulfate

A total of 50,917 SMs divided into 11 libraries were screened against *C. jejuni* 81–176, to identify potential inhibitors of the Tat system (Supplementary Table S2). The hits were identified by screening in the presence of 1 mM CuSO_4._ Since CueO contributed to CuSO_4_ resistance and its function requires Tat system transport, increased susceptibility to copper was used as an indicator during high-throughput sequencing for Tat-specific SM inhibitors. The growth profile obtained for each SM tested was compared with the growth of the Δ*tatC* mutant (susceptible to 1 mM CuSO_4_; no OD_600_ increase over time) and *C. jejuni* 81–176 (resistant to 1 mM CuSO_4_; Supplementary Figure S1) in the presence of 1 mM CuSO_4_. Out of them, 679 SMs completely inhibited the growth of *C. jejuni* in the presence of 1 mM copper sulfate after comparison of the turbidimetric values obtained with the *C. jejuni ΔtatC* mutant. The number of SM identified per library was not proportional to the concentration of the libraries (ranging between 2.5 and 12.5 μg/mL). However, known bioactive collection (Biomol 4, MSDiscovery 1, Microsource 1, and MIH Clinical Collection 1 and 2) displayed higher hit rate (approximately 3.54%) than commercial libraries (Asinex, Chembridge 3, Chemdiv 4, Enamine 2, Lifechemicals 1, and Maybridge 5; approximately 1.20%), suggesting that each library might be composed of SM with distinct chemical structures (Supplementary Table S2). The average hit rate across all libraries was 1.33%, which is significantly higher than the optimal hit rate proposed by the NSRB screening guidelines (0.3% or approximately 1 hit/plate) (Drozd, 2012). Out of the 679 SMs identified, 177 SMs had no predicted bioactivity in eukaryotic cells (i.e., selected SM had no predicted interactions with known eukaryotic targets based on *in silico* counter screens) and followed the Lipinski rule of five based on *in silica* analyses (Lipinski et al., 2001; Lipinski, 2004). Among the 177 SMs, 66 SMs had a thiourea group, 46 SM had a benzimidazole group, 38 SM had an acylhydrazone group, and 11 SM had an oxadiazole group. These 177 SMs (107 SM from Asinex, 28 SM from Chembridge, 17 SM from Life chemicals, and 25 SM from Maybridge) were selected for the secondary screen upon resynthesis.

### 21 SMs increased the susceptibility of *Campylobacter jejuni* to CuSO_4_ and reduced FDH activity *in vitro*

**Figure 1:**
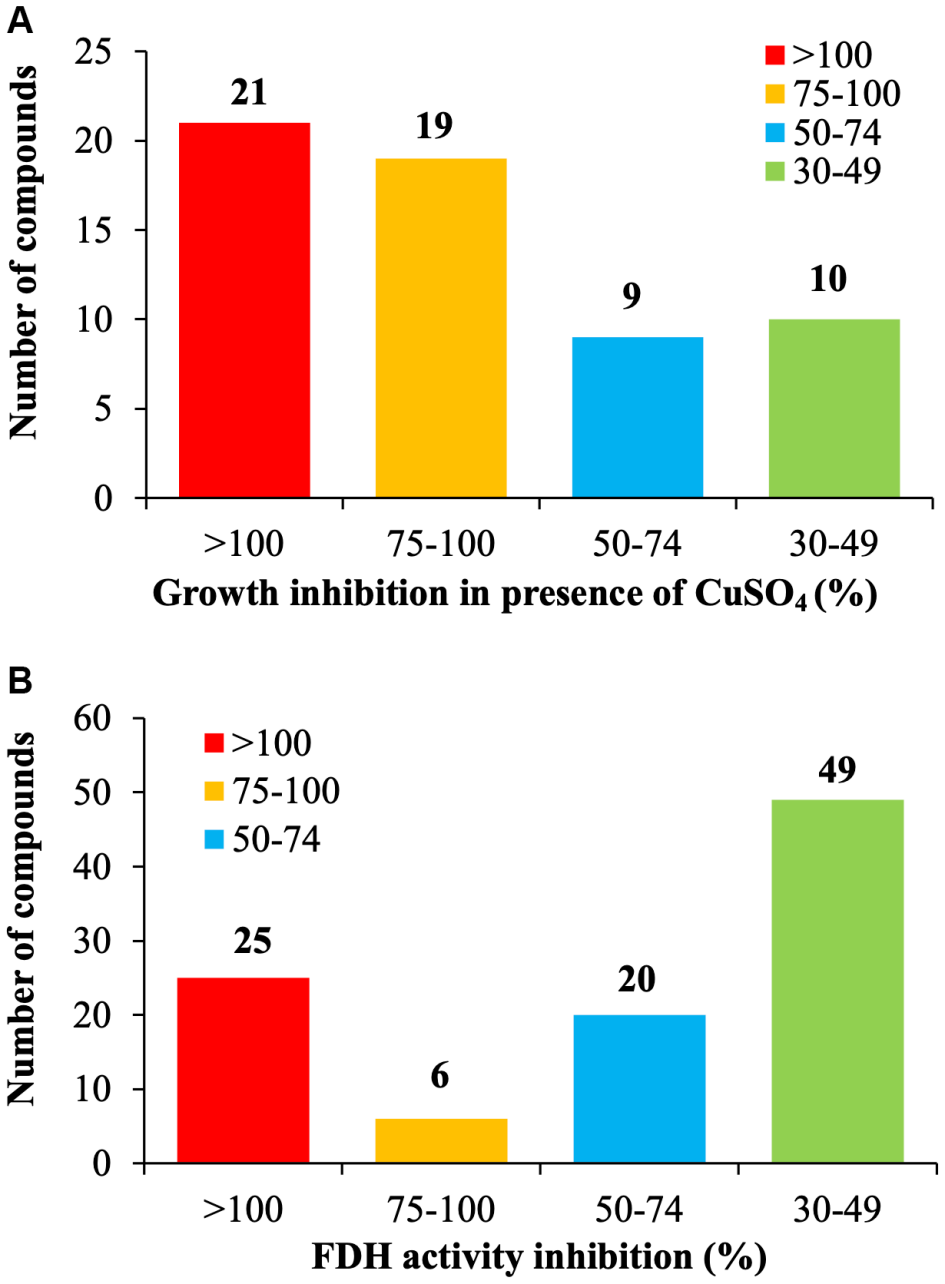
Identification of Tat dependent inhibitors in *C. jejuni* 81–176. **(A)** Copper sulfate sensitivity assay. *C. jejuni* 81–176 was challenged for 24 h with 6.25 μg/mL of SM plus 0.5 mM CuSO_4_ in microaerophilic condition. The growth inhibition was determined by measuring the optical density and being compared to the DMSO control. **(B)** Formate Dehydrogenase (FDH) inhibition activity assay. *C. jejuni* 81–176 was challenged for 24 h with 6.25 μg/mL of SM in microaerophilic condition. The FDH activity was determined by measuring the optical density and being compared to the DMSO control. *N* = 3 replicates per group.

Of the selected 177 SMs tested, only 33.3% of the SM (*n* = 59/177) reduced at least 30% growth of *C. jejuni* in the presence of 0.5 mM CuSO_4_ compared with the DMSO control (Figure 1A). Twenty-one SM completely inhibited C. jejuni growth, 19 SM reduced the growth of *C. jejuni* by 75 to 99%, and 19 SM reduced the growth of *C. jejuni* between 30 and 75% in the presence of 0.5 mM CuSO_4_ compared to the DMSO control. The Fdh inhibition activity assay showed that 56.5% of the SMs (*n* = 100/177) reduced *C. jejuni* Fdh activity by 30 to 100% compared with the DMSO control (Figure 1B). Overall, 21 SMs that sensitized *C. jejuni* to copper sulfate also displayed a significant reduction in FDH activity (>30%), suggesting that these SMs affect the growth of *C. jejuni* in a Tat-dependent manner. These 21 SMs were selected for further testing.

The inhibitory activity of the 19/21 SM (2 SM could not be resynthesized) was also tested on other *Campylobacter* strains (*n* = 11; Supplementary Table S1) and commensal/beneficial gut bacteria (*n* = 7) using a copper sensitivity assay at 0.5 mM CuSO_4_ with 6.25 μg of SM_,_ as mentioned above. Overall, 19 SMs completely inhibited the growth of 12 *Campylobacter* strains in the presence of copper sulfate while having minimal impact on the growth of two commensal/beneficial gut bacteria in a similar growth condition (growth inhibition in the presence of copper sulfate lower than 50% compared with the DMSO control). The selected 19 SMs displayed a very similar spectrum of activity profiles. They completely inhibited the growth of all *Campylobacter* strains tested at 6.25 μg. Furthermore, the 19 SMs had no growth effect on *Escherichia coli* Nissle 1917, *Streptococcus bovis, Bifidobacterium adolescentis, Bifidobacterium longum,* and *Bacteroides thetaiotaomicron* at 6.25 μg in the presence of 0.5 mM CuSO_4_; however, they affected the growth of *Lacticaseibacillus rhamnosus* GG (*n* = 13 SM), *Enterococcus faecalis* (*n* = 16 SM), and *Levilactobcillus brevis* (*n* = 2 SM; Table 1).

### Selection of eight most potent Tat-dependent inhibitors

**Figure 2:**
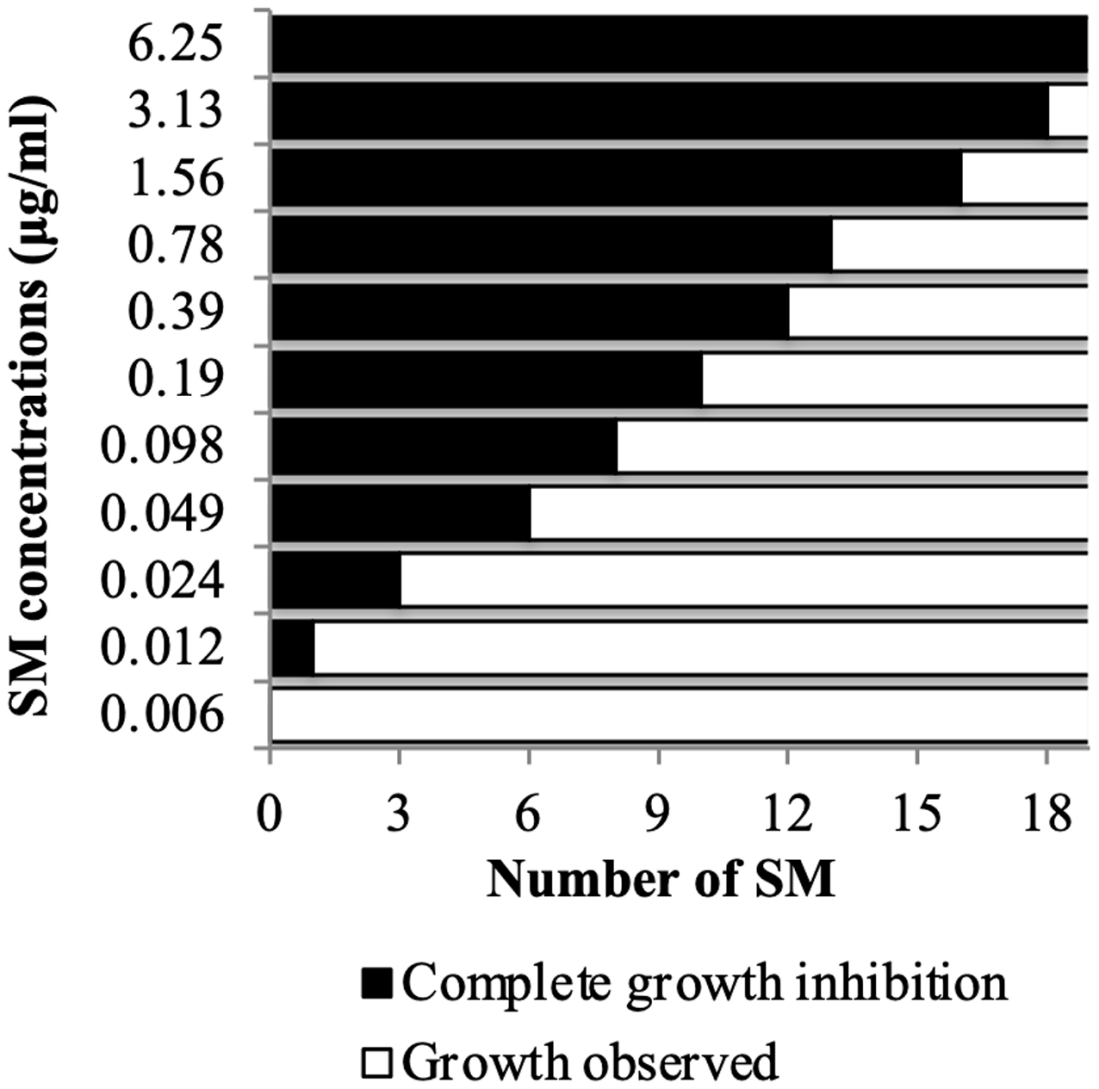
Copper sulfate sensitivity dose–response assay *in vitro*. *C. jejuni* 81–176 was challenged for 24 h with SM concentration ranging between 0.006 and 6.25 μg/mL in presence of 0.5 mM CuSO_4_. A total of 19 compounds were tested. *N* = four replicates per group.

A copper sulfate sensitivity dose–response assay was performed *in vitro* on *C. jejuni* 81–176 using the selected 19 Tat-dependent inhibitors (Figure 2). One SM had an MIC of 3.13 μg/mL; two SMs had MIC at 1.56 μg/mL; three SMs had MIC at 0.78 μg/mL; one SM had MIC at 0.39 μg/mL; two SMs had MIC at 0.19 μg/mL; two SMs had MIC at 0.098 μg/mL; two SMs had MIC at 0.049 μg/mL; three SMs had MIC at 0.024 μg/mL; and one SM had MIC at 0.012 μg/mL in the presence of 0.5 mM CuSO_4_. Interestingly, the number of strains affected by the compounds (i.e., spectrum of activity) was not correlated with their antimicrobial efficacy (e.g., MIC and MBC).

**Figure 3:**
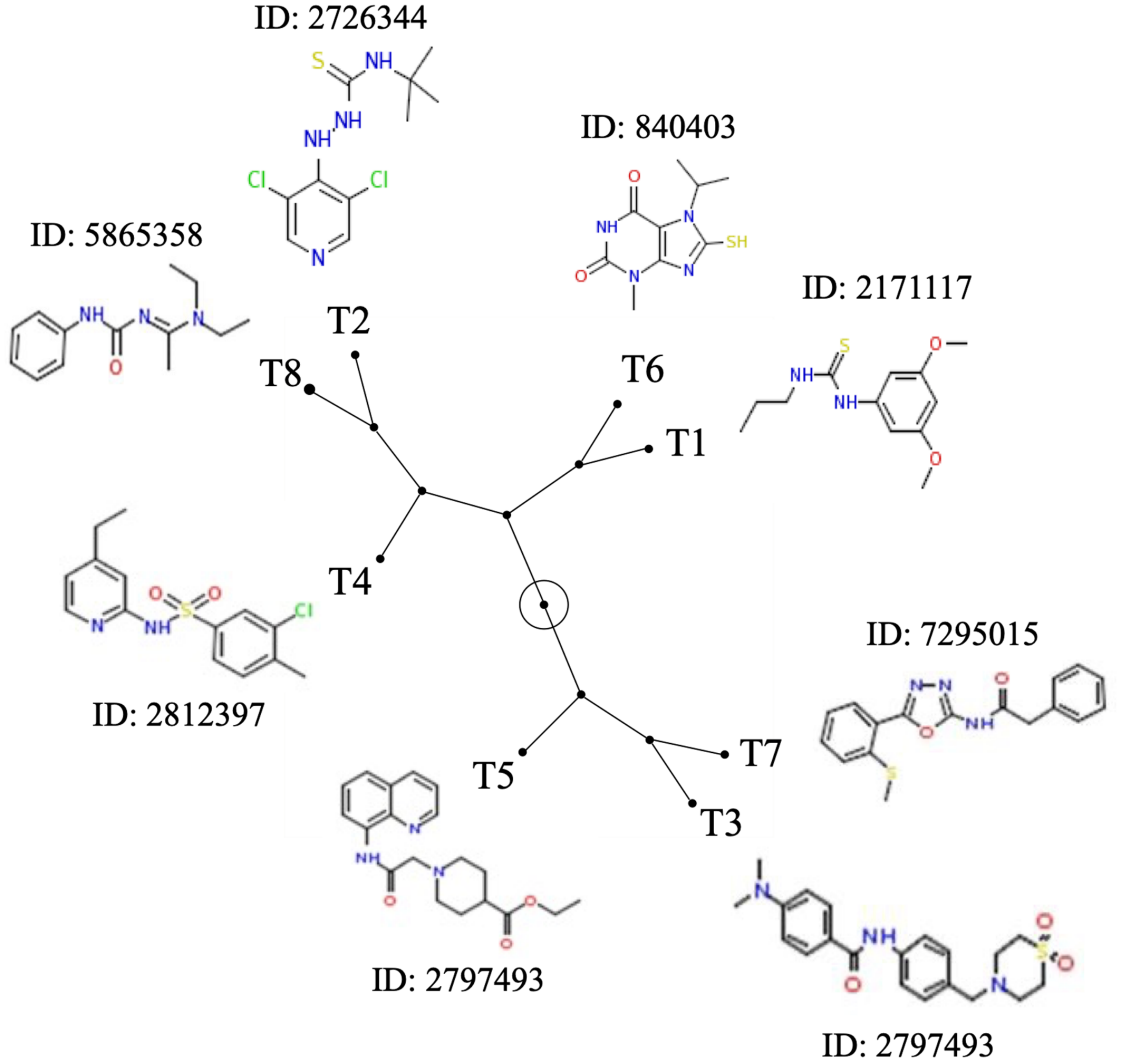
Chemical structure diversity of the eight most potent Tat-dependent inhibitors. The constellation tree was built based on the structure similarity score generated based on 3D Tanimoto scoring method in PubChem (https://pubchem.ncbi.nlm.nih.gov/assay/assay.cgi?p=clustering). The circled node represents the root of the tree. Each SM is associated with its chemical structure and its PubChem ID.

Based on the antimicrobial activity (i.e., efficacy and spectrum of activity) and *in silica* data obtained, we selected eight SMs with high drug-like properties, little or no growth effect on commensal/beneficial gut bacteria, and high efficacy against several *Campylobacter* strains (Table 1; Supplementary Table S1). Overall, the antimicrobial efficacy of the selected eight SMs was similar across the 12 *Campylobacter* strains tested with MIC values of 0.01 μg/mL and higher, and MBC values of 0.04 μg/mL and higher (Supplementary Tables S4, S5, respectively). In addition, the three-dimensional analysis of the chemical structure of the eight selected SMs showed that T8 and T2, T6 and T1, and T7 and T3 displayed high structural similarities, while T4 and T5 had unique chemical structures (Figure 3). Furthermore, most of the SMs have sulfur and/or nitrogen-based functional groups (thiourea, imidazole uracil, sulfonamide, thiomorpholine dioxide, phenylurea, pyridine, piperidine, oxadiazole, and quinoline). However, no association was detected between the chemical structure of the SM and their antimicrobial properties.

### The eight Tat-dependent inhibitors reduced *Campylobacter jejuni* intracellular population In infected colon epithelial cells with low cytotoxicity level

**Figure 4:**
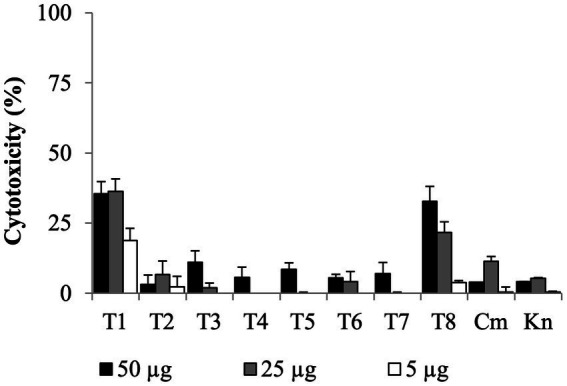
Dose-dependent cytotoxicity assay of the eight Tat-dependent inhibitors using colon epithelial (Caco-2) cells. Bar: standard deviation; Cm, chloramphenicol; Kn, kanamycin; LDH, lactate dehydrogenase; *N* = four replicates per group.

All SMs completely cleared internalized *C. jejuni* 81–176 in Caco-2 cells after 24 h of treatments with a concentration of SM ranging from 0.63 μg/mL to 10 μg/mL (Table 1). Most of the SMs displayed low toxicity (at most 10%) to Caco-2 cells when treated with 50 μg/mL for 24 h (Figure 4). The toxicity values were comparable to the kanamycin and chloramphenicol-treated cells. Only T1 and T8 displayed toxicity level (36 and 33%, respectively) when treated with 25 μg/mL or 50 μg/mL of SM for 24 h; however, T1 and T8 cleared intracellular *C. jejuni* 81–176 at 0.63 μg/mL and 5 μg/mL, respectively, which represent concentrations up to 80-fold lower compared with ones used for the toxicity assay. Overall, T1, T2, T7, and T8 displayed the most promising antimicrobial properties *in vitro* were selected for further analyses.

### T2 and T7 treatments reduced *Campylobacter jejuni* load in colonized chicken ceca

**Figure 5:**
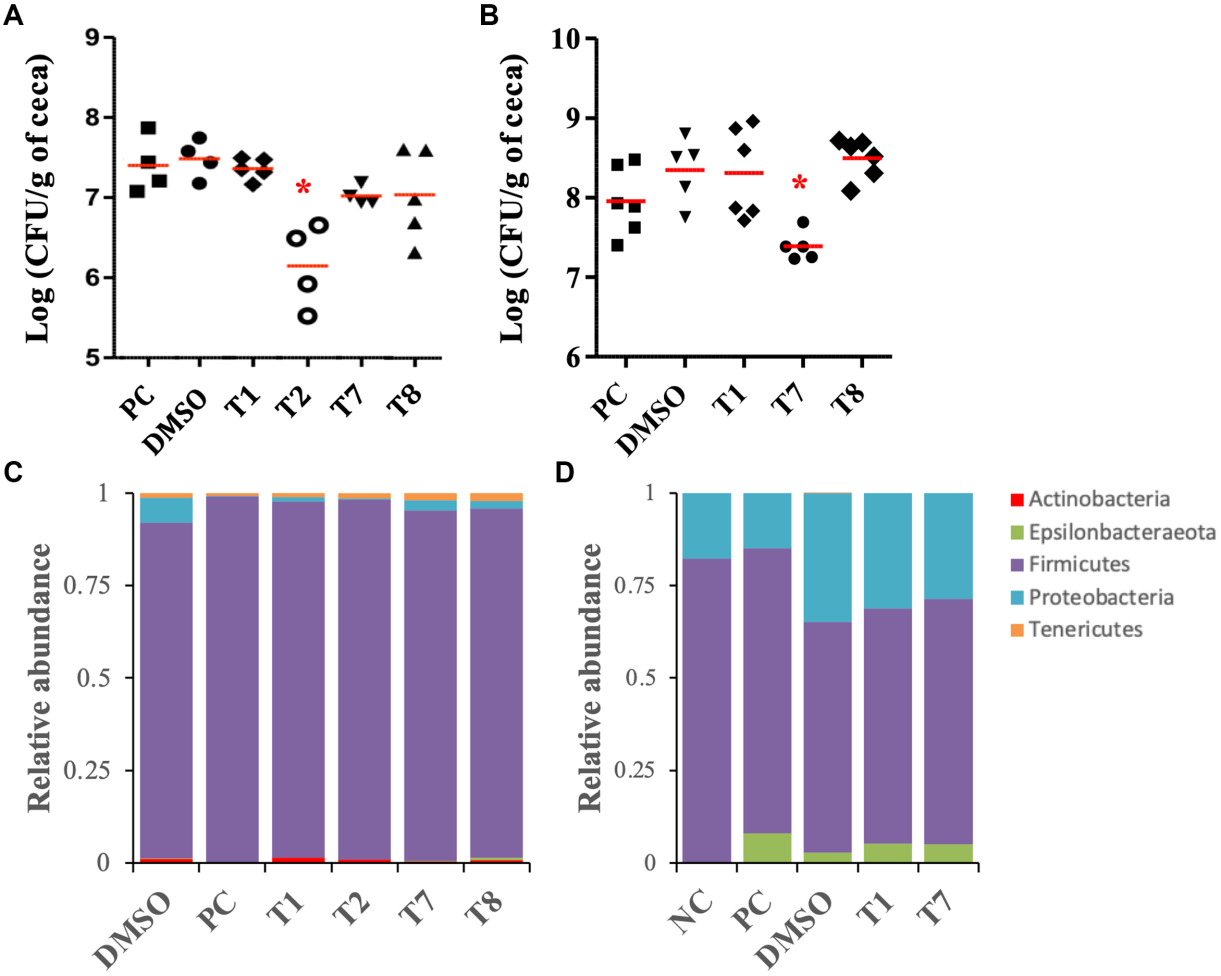
Effect of the four most potent Tat-dependent inhibitors on the persistence of *C. jejuni* in three- and five-week-old chicken ceca and its microbiota. **(A)** *C. jejuni* abundance in five-week-old chicken ceca after SM treatment (*n* = 5–4 chickens per group). The *C. jejuni* population in ceca was determined after 5 days of treatment with 0.127 mg/mL of SM. Each dot represents a chicken. Red bar represents the mean. *: significant reduction of the *C. jejuni* population in ceca compared to the DMSO control group (*p* < 0.01). **(B)** *C. jejuni* abundance in three-week-old chicken ceca after SM treatment. The *C. jejuni* population in ceca was determined after 5 days of treatment with 0.255 mg/mL of SM (*n* = six to five chickens per group). **(C)** Relative abundance at the phylum level in five-week-old chicken ceca after treatment. **(D)** Relative abundance at the phylum level in three-week-old chicken ceca after treatment. NC: not inoculated not treated chickens; PC: colonized not treated chickens; DMSO, T1, T2, T7, T8: colonized chickens treated with DMSO or one of the selected SM.

As a proof of concept, the selected SMs (T1, T2, T7, and T8) were tested on 5-week-old chickens inoculated with a mixture of *C. jejuni* strains (Supplementary Table S1), to assess the clearance of *C. jejuni* in chicken right before the slaughter. Following 5 days of SM treatment (twice a day, approximately 0.225 mg of SM per kg body weight per treatment), colonized chickens treated with T2 displayed a significant reduction (1.2-log) in *C. jejuni* population per gram of cecum compared with the DMSO control group, which harbored approximately 5×10^7^ CFU per gram of cecum (*p* < 0.01; Figure 5A). Colonized chickens treated with the T7 and T8 groups displayed a 0.5-log reduction in *C. jejuni* population in ceca compared with the DMSO control group (*p* > 0.05), while the T1 treatment did not reduce the abundance of *C. jejuni* in ceca compared with the DMSO group. No significant difference in body weight was recorded between treatment groups before or after 5 days of treatment (*p* > 0.05; Supplementary Figure S2A).

The antimicrobial activity of T1, T7, and T8 was also tested on 3-week-old chickens inoculated with a cocktail of *C. jejuni* strain (Supplementary Table S1), to assess the clearance of *C. jejuni* in young chicken right after inoculation. T2 could not be resynthesized in sufficient quantities and, thus, was not tested on this 3-week-old experiment. Following 5 days of SM treatment (approximately 0.127 mg of SM per kg body weight per treatment), 3-week-old chickens treated with T7 displayed a significant reduction (0.9-log) in *C. jejuni* population per gram of cecal contents compared with the DMSO-treated group, which harbored approximately 4 × 10^8^ CFU per gram of cecal contents (*p* < 0.01; Figure 5B). Chickens treated with T1 and T8 harbored similar *C. jejuni* abundance in ceca compared with the DMSO control group. No significant difference in body weight was recorded between treatment groups before or after 5 days of treatment (*p* > 0.05; Supplementary Figure S2B).

### The SM treatments had minimal impact on the chicken cecal microbiota

The impact of the SM treatments on the cecal microbiota of the 3 and 5-week-old chickens was studied using 16S sequencing. After processing and taxonomic assignment with the SILVA reference database, 682,777 sequences were obtained from the 58 samples studied. Sequencing depth varied between 6,648 and 15,749 reads per sample (mean = 9,753 reads per sample). Cecal samples were normalized to 6,600 sequences per sample for the data presented below.

For the 3-week-old chicken experiment, the analysis of the cecal alpha diversity, using Faith’s PD and Shannon diversity index, indicated no significant differences between the treatment groups. On the other hand, the DMSO treatment (DMSO, T1, T2, T7, and T8 groups) significantly increased the Shannon index value (approx. 6.3) compared with the colonized, non-treated groups (PC; approx. 5.5; *p* < 0.04). The beta diversity analysis demonstrated that the SM treatments (T1, T2, T7, and T8 groups) had minimal impact on the global microbiome composition compared with the DMSO group (Supplementary Figure S3A). Most of the variations were detected between the colonized chickens treated with DMSO (DMSO, T1 and T7 groups) and the chickens that were not treated with DMSO (NC and PC groups; *p* < 0.01). Additional details concerning the impact of the DMSO and *C. jejuni* colonization on the cecal microbiome at the genus and species levels are shown in Supplementary Figures S3A–C. Overall, the cecal microbiome was composed of Firmicutes (90–99%), followed by Proteobacteria (0.1–6.7%), Tenericutes (0.6–2.1%), Actinobacteria (0.06–1.4%), and Epsilonbacteraeota (0.02–0.1%, Figure 5C). No significant differences were detected at the phylum level between the SM-treated groups (T1, T2, T7, and T8) and the associated control group (DMSO). At the species level, T8 treatment significantly increased GCA-900066225 (11.6-fold), and T7 treatment significantly increased Ruminococcaceae UCG-014 (2.2-fold) compared with the DMSO group (*p* < 0.01).

For the 3-week-old chicken experiment, the analysis of the cecal alpha diversity, using Faith’s PD and Shannon diversity index, indicated no significant differences between the treatment groups of (Supplementary Figures S3D,E). Similarly, beta diversity analysis, using the weighted uniFrac, confirmed that the SM treatments (T1 and T7 groups; T8 was not included for microbiota study since it did not have positive impact on *C. jejuni* load in the cecum) had minimal impact on the global microbiome composition compared with the DMSO group (Supplementary Figure S3F). Most of the variations were detected between the colonized chickens treated with DMSO (DMSO, T1 and T7 groups) and the chickens not treated with DMSO (NC and PC groups; p < 0.01). Additional details concerning the impact of DMSO and inoculation of *C. jejuni* on the cecal microbiome at the genus and species levels are shown in Supplementary Figures S3D–F. Overall, the cecal microbiome was composed of Firmicutes (62–82%), followed by Proteobacteria (15–35%) and Epsilonbacteraeota (0–8%, Figure 5D). No significant differences were detected at the phylum level between the SM-treated groups (T1 and T7) and the associated control group (DMSO). At the species level, *Caproiciproducens* (6.5-fold and 4.4-fold, respectively) and *Flavonifractor* (1.7-fold) were significantly higher in T1 and T7 groups compared with the DMSO group. *Eubacterium coprostanoligenes* group (8-fold) and *Neglecta timonensis* (only detected in the T7 group at 0.8%) were significantly higher in the T7 group compared with the DMSO group (*p* < 0.01).

### Docking studies predicted that T2 and T7 interact with the TatC protein of the Tat system

**Figure 6:**
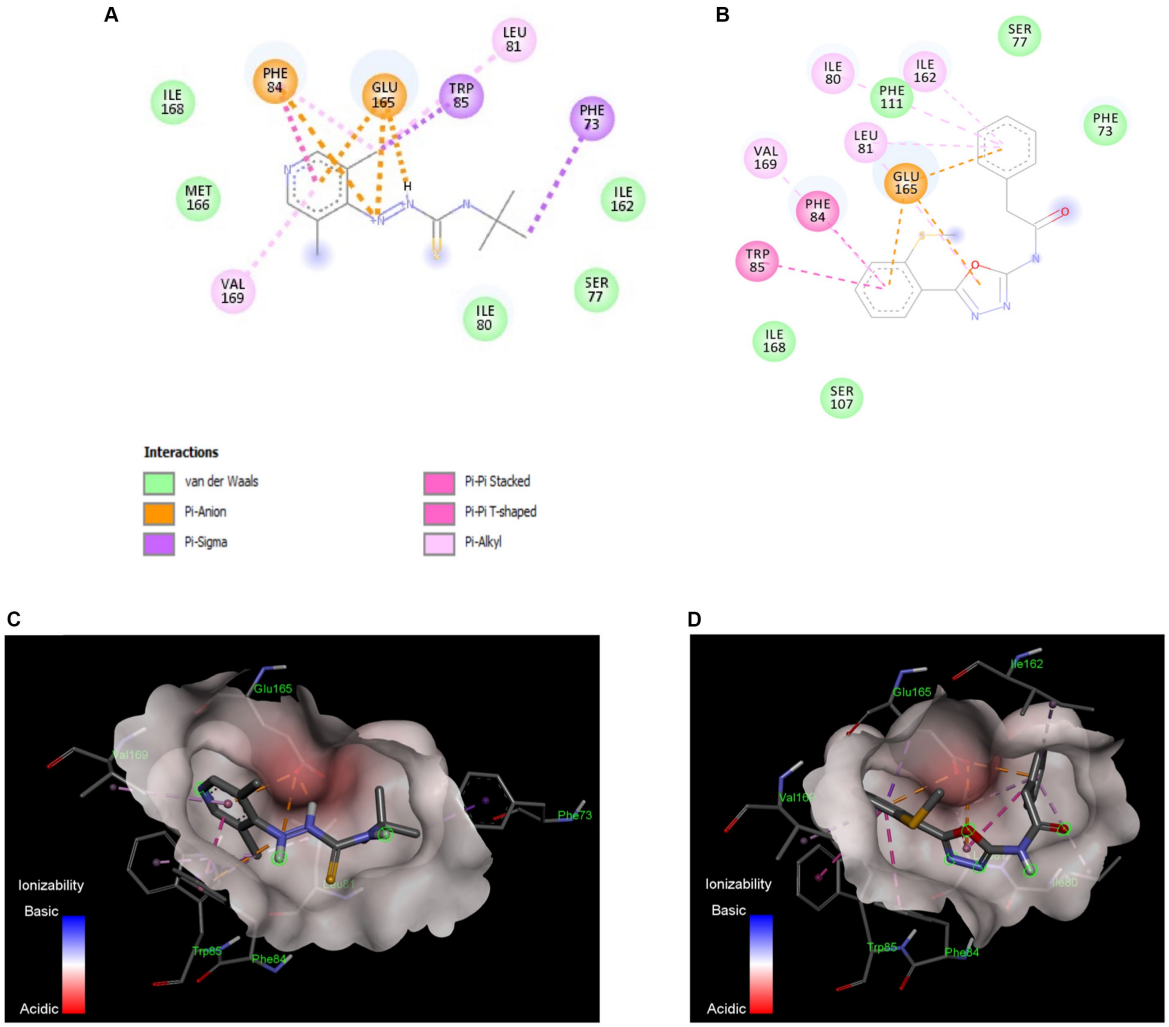
*In silico* docking model between the Tat inhibitors and the TatC system in *C. jejuni*. Binding interactions of the most active small molecule inhibitors with a homology model of TatC from *Aquifex aeolicus*, Compound T2 **(A)**, Compound T7 **(B)**. The compounds bind in the same pocket and the interaction with key residue Glu165 and Trp85 is responsible for Tat C inhibition. The ionizability model for T2 **(C)** and T7 **(D)** of residues in the binding pocket indicates the Pi-anion interactions with Glu165 and Pi-Pi interaction with Trp85. These docking models indicate that TatC inhibition may be responsible for the anti-*C. jejuni* activity of T2 and T7.

TatC subunit is the largest and most important part of Tat system, while Tat A and Tat B are much smaller units. The docking studies were conducted with all Tat subunits; however, TatA and B protein folding could not be predicted with high certainty due to smaller protein size. Therefore, we focused on TatC, which was modeled with a high degree of predictability and well-defined binding pockets, giving repeatable docking results. The *in silico* docking study demonstrated that both active compounds T2 and T7 bind in the same hydrophobic binding pocket associated with the key active residue Glu 165 (Figure 6). The calculated binding energy for T2 is −6.26Kcal/mol, while T7 has a higher binding energy of −8.0 Kcal/mol. The T2 thiourea nitrogen forms a hydrogen bond with Ile80, while the phenyl ring forms Pi-Pi stacking interaction with Phe87. Trp85 and Phe87 form Pi-sigma interaction with the T2 pyridine ring and tert-butyl group, respectively (Figures 6A,C). In addition, there are several hydrophobic interactions within carbon chains of T2 and Ile80, Ser 77, Ile162, Phe111, Ser107, Ile168, Met166, Gln83, and Ser17. The T7 functional groups (i.e., oxadiazole and phenyl groups) bind to several amino acids that are located inside the hydrophobic core of TatC (Figure 6B). More precisely, all aromatic rings of T7 have π-anion interactions with the key Glu165 (Figures 6B,D); the phenylacetamide group has π-alkyl interactions with Val169, π-π interactions with Phe84 and Trp85, and van der Waals interactions with Ile168 and Ser107; the methylthio-phenyl group has π-alkyl interactions with Ile80, Ile162, and Leu81, and van der Waals interactions with Phe111, Ser77, and Phe73. π- Σ interactions are also detected on the amide and methanethiol groups. Thus, anti-*C. jejuni* activity of both T2 and T7 may be attributed to their binding to hydrophobic pocket in TatC and the interaction with key residues Glu165 and Trp85 (Figure 6).

**Figure 7:**
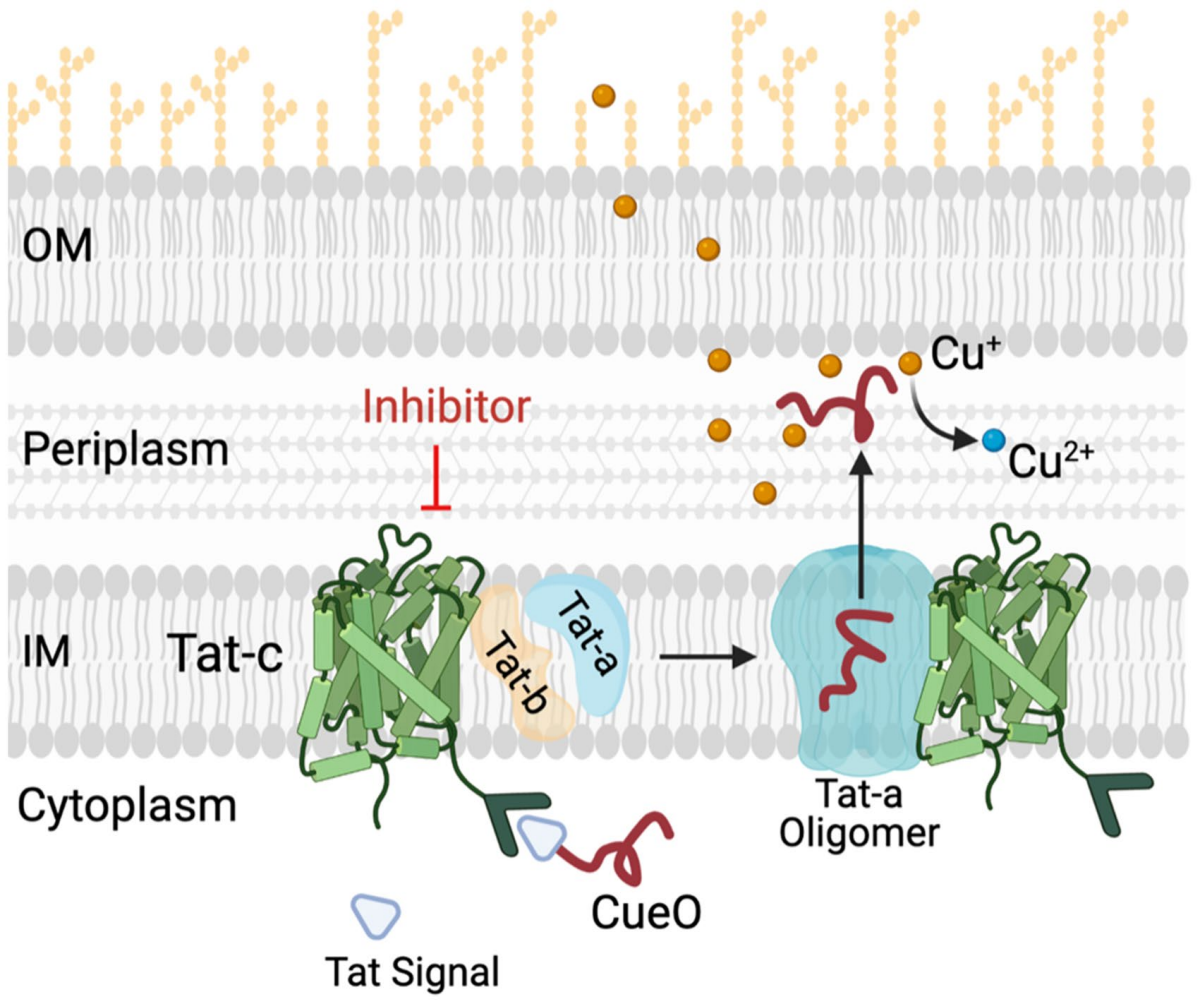
Role of Tat system in copper (Cu) homeostasis in *C Jejuni*. *C jejuni* employs a Tat complex (Tat-A, B, C) for the transport of proteins from cytoplasm to periplasm. TatC is the core transmembrane component of this complex located in inner membrane (IM), responsible for translocation of folded proteins such as multi-copper oxidase (CueO) and formate dehydrogenase (Fdh) from cytoplasm to periplasm. CueO is critical for oxidation of Cu^+^ which are highly toxic as compared to relatively non-toxic Cu^2+^ form. Thus, in the presence of TatC inhibitors the transportation of important cytoplasmic proteins such as CueO and Fdh is hampered. This results in increased sensitivity of *C jejuni* to copper. OM and IM, outer and inner membrane, respectively; CueO, copper oxidase; Cu, copper.

## Discussion

The emergence of antibiotic-resistant isolates significantly reduces the antimicrobial efficacy of current control methods which are used to mitigate human campylobacteriosis (Kaakoush et al., 2015; Subbiah et al., 2016; Yang et al., 2019). Previous studies showed that the Tat system is highly conserved in *Campylobacter* spp. and is critical for the persistence of *Campylobacter* in the intestinal tract of poultry, a recurrent reservoir for *Campylobacter* (Rajashekara et al., 2009; Hitchcock et al., 2010; Hermans et al., 2011). Additionally, the absence of homologous proteins in chickens and mammals increases the likelihood that the Tat system-based control strategies would avoid damage or unwanted interaction with host cells (Kassem and Rajashekara, 2014). Previous studies demonstrated increased copper sulfate sensitivity and reduced Fdh activity in *Campylobacter* when the Tat system was non-functional; therefore, these two indicators were used to select the compounds that are likely to specifically inhibit the Tat system (hit compounds). A total of 50,917 SMs distributed among 11 “commercial” or “known bioactive” libraries provided by ICCB-Longwood version v2010.10.29 and v2012.01.26 were screened. A total of, eight SMs (T1-T8; 10 ng/mL and higher) sensitized several *C. jejuni* and *C. coli* isolated from human and poultry to sublethal dose of copper sulfate (0.5 mM) *in vitro*, and had minimal to absent toxicity in Caco-2 colon epithelial cells. These initial findings support our hypothesis that the Tat system is a promising target for the development of the anti-*C. jejuni* control method with minimal impact on eukaryotes. Further efforts will validate the molecular target of T2 and T7 to facilitate their uses against *C. jejuni* infections in humans. Distinct chemical backbones were observed among the eight SMs, suggesting that multiple chemical scaffolds can target the Tat system in *C. jejuni* or interact with chaperoning proteins. Most of the SMs were characterized by sulfur and/or nitrogen-containing functional groups (thiourea, imidazole uracil, sulfonamide, thiomorpholine dioxide, phenylurea, pyridine, piperidine, oxadiazole, and quinoline). Furthermore, SM with low molecular weights (< 400 Da) completely cleared intracellular *C. jejuni* between 0.63 μg/mL and 2.5 μg/mL, while SM with high molecular weights (> 400 Da) completely cleared intracellular *C. jejuni* at 5.0 μg/mL and higher. Therefore, the molecular weights could serve as a selection criterion of SM for testing them in a cell culture setting (Schuffenhauer et al., 2006).

This study identified two potential Tat inhibitors (T2 and T7) that significantly reduced the cecal *C. jejuni* load (up to 1.2-log) in colonized chickens after a 5-day treatment twice a day with 0.225 mg or 0.127 mg of SM per kg body weight per treatment, respectively. Furthermore, the SM treatments did not affect the chicken body weight gain or disturb the cecal microbiota. This preliminary *in vivo* data validated that the Tat system could represent a good target for the development of novel and safe control methods against *C. jejuni*. Future studies will focus on the development of T2 and T7 derivatives with improved antimicrobial efficacy, dosage titration, and testing these compounds in farm-like settings. Similarly, the addition of our SM to the feed will be tested, which is the preferred delivery method in commercial poultry operations. Since the solvent used to deliver the SM to the chickens had a significant impact on the cecal microbiota, future studies will focus on the development of water-soluble T2 and T7 derivatives, optimizing the dosage and bioavailability for these molecules in chicken tissues after treatment.

Differences in antimicrobial efficacy were observed with T7 and T8 between the 3 and 5-week-old chicken experiments. These differences might be due to SM dosage used and/or the microbial composition in chicken ceca. Interestingly, the abundance of the *Eubacterium coprostanoligenes* group was significantly increased in 3-week-old chickens treated with T7 compared with the DMSO-treated chickens. *Eubacterium coprostanoligenes* is an anaerobe genus involved in the reduction of cholesterol (Wei et al., 2020). However, it was also showed that depletion in the membrane cholesterol was associated with a reduction in *C. jejuni* cytolethal distending toxin-induced pathogenesis and, thus, attenuated the intoxication of host cells (Lin et al., 2011; Lai et al., 2013, 2015). Future studies will assess whether this bacterium has anti-*C. jejuni* properties. If true, the combination of our Tat inhibitor T7 with *Eubacterium coprostanoligenes* will also be investigated to mitigate *C. jejuni* in poultry. It was also observed that treating chickens with DMSO, using oral gavage, significantly affected the composition of the chicken ceca microbiota (*Lactobacillus*, *Romboutsia*, *and Enterobacteriaceae*). *Enterobacteriaceae* and *Lactobacillaceae* bacteria are major components of the initial intestinal microbiota and are essential for the installation and proliferation of aerobic sensitive bacteria over time (Lin and Zhang, 2017). Furthermore, certain *Lactobacillus* isolates have been shown to harbor potential antagonistic properties against *Campylobacter* in chicken (Dec et al., 2018). Therefore, the microbiome alterations caused by the DMSO treatment might have enhanced the antimicrobial efficacy of our selected compounds.

The *in silico* docking study demonstrated that our Tat inhibitors T2 and T7 bind with a key amino acid residue, Glu 165, are located in the hydrophobic core of TatC. Glu 165 is conserved as polar glutamine or glutamate across species of bacteria and plays an important role in TatC functioning. Glu 165 forms a hydrogen bonding network with Ser107 and Trp 85, which is important for generating electrochemical gradient for Tat energy function (Rollauer et al., 2012). Trp 85 forms an important interaction between Tat signal and W85G and suppresses Tat signaling and transportation (Ramasamy et al., 2013). It is also shown that TatC forms a glove- like shape, and Glu 165 sits in the concave surface, where it can interact with TatA (Figure 7). The ionized Glu 165 present in hydrophobic core of concave TatC is important for interactions with TatA. The mutation E165A severely compromises TatC functioning. Thus, our *in silico* data suggest that the binding of T2 and T7 in Glu-165 hydrophobic pocket of TatC might perturb the transport of CueO and Fdh. It is important to mention that the model displayed in this study to assess the binding affinity of our SM to TatC was conducted using the TatC crystal structure from *Aquifex aeolicus*. No crystal structure of other TatC proteins is available to date. Thereby, no predictive docking modeling of our SM with other TatC protein could have been conducted in this study to assess the specific binding of our SM to *C. jejuni* TatC. However, TatC is the most conserved protein of the Tat system across bacteria (i.e., *E. coli*, *Aquiflex aeolicus*, *C. jejuni*, *Thermus thermophilus*, and *Staphylococcus aureus*) (Lee et al., 2006; Ramasamy et al., 2013; Patel et al., 2014). Based on a NCBI Blastp analysis conducted on 01/10/2024, it was found that *C. jejuni* TatC displays at least 98.37 and 93.88% sequence similarity with other *C. jejuni* TatC sequences and *C. coli* TatC sequences (*n* = 100 sequenced tested per species), respectively. These observations support the antimicrobial efficacy of our SM against the *C. jejuni* and *C. coli* strains tested in this study. On the other hand, low sequence similarity was observed between *C. jejuni* TatC and the few TatC obtained from other non-thermophilic *Campylobacter* strains; *Campylobacter fetus* (similarity of 59.43% and above; *n* = 14), candidatus *Campylobacter infans* (similarity of 55.95%; *n* = 2), *Campylobacter hyointestinalis* (similarity of 61.76% and above; *n* = 12) and *Campylobacter upsaliensis* (similarity of 72.02% and above; *n* = 48). Future studies will investigate whether our best candidates could be also used against non-thermophilic *Campylobacter in vivo* or if better candidates can be identified from the initial libraries tested in this study. Furthermore, a similarity of 34.55 and 26.73% were observed between TatC obtained from *C. jejuni* and *Escherichia coli* Nissle 1917 and *Bifidobacterium longum*, respectively. To date, no TatC proteins were identified in *L. brevis* and *rhamnosus L.*, *Bifidobacterium adolescentis* and *lactis*, and *Enterococcus faecalis*. These *in silico* results concord with the limited impact of our SM on commensal/beneficial bacteria.

In conclusion, data presented in this study represent a proof of concept that the Tat system represents a good target for the development of novel and safe control methods against *C. jejuni*. We have identified two Tat-dependent inhibitors (T2 and T7) with the potential to become effective control method to mitigate *C. jejuni* in poultry production systems. However, additional efforts are required to improve the antimicrobial efficacy of the Tat compounds before being used to control *C. jejuni* in large-scale poultry production systems.

## Data availability statement

The datasets presented in this study can be found in the NCBI repository, accession number PRJNA1023035.

## Ethics statement

The animal study was approved by Institutional Animal Care and Use Committee (IUACUC) protocol n° 2010A00000149-R2-AM1. The study was conducted in accordance with the local legislation and institutional requirements.

## Author contributions

LD: Data curation, Formal analysis, Investigation, Methodology, Validation, Visualization, Writing – original draft, Writing – review & editing. MD: Conceptualization, Methodology, Validation, Writing – review & editing. AK: Conceptualization, Methodology, Validation, Writing – review & editing. JA: Methodology, Writing – review & editing. JF: Conceptualization, Investigation, Methodology, Supervision, Writing – review & editing. RK: Formal analysis, Methodology, Validation, Visualization, Writing – review & editing. GR: Conceptualization, Funding acquisition, Investigation, Methodology, Project administration, Resources, Supervision, Validation, Writing – review & editing. YH: Investigation, Methodology, Writing – review & editing.

## Footnotes

^1^ https://mcic.osu.edu/
