# Supplementary material for: Identification of novel small molecule inhibitors of twin arginine translocation (Tat) pathway and their effect on the control of Campylobacter jejuni in chickens
Source: Front Microbiol. 2024 Apr 17;15:1342573. doi: 10.3389/fmicb.2024.1342573 (PMC11061419; doi:10.3389/fmicb.2024.1342573)
Supplement: Supplementary file 1 [file Data_Sheet_1.pdf]

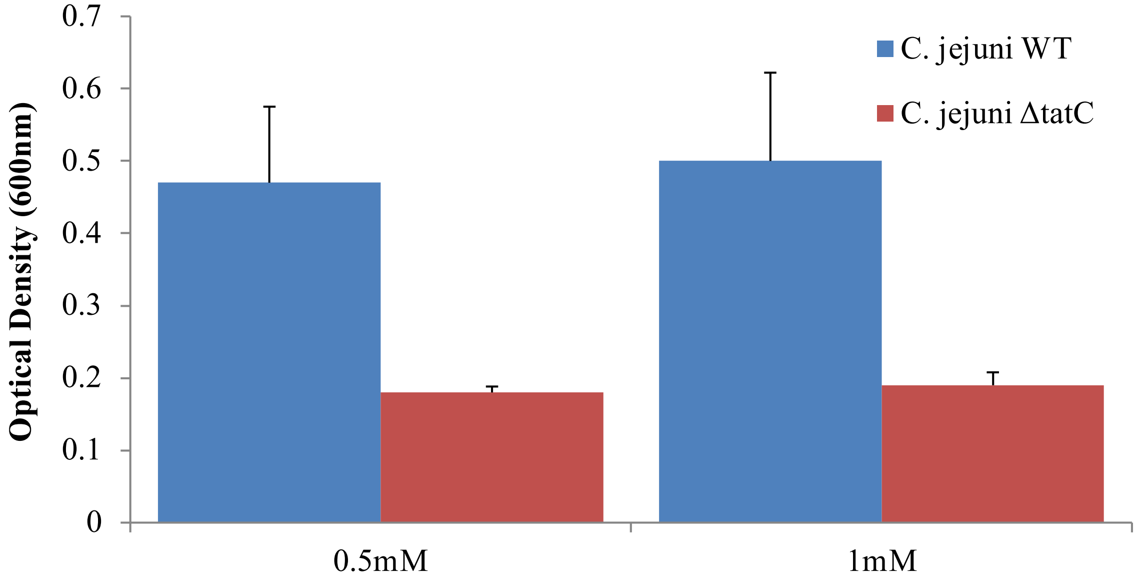

Supplemental Figure 1. Growth of *Campylobacter jejuni* 81-176 and ΔtatC mutant in presence of copper sulfate after 24 hrs post-incubation. *C. jejuni* was grown in MH broth supplemented with 0.5 or 1 mM of copper sulfate at 42ºC in microaerophilic conditions. Optical density (600 nm) was measured at 0 and 24 hrs post-incubation. Bar: standard deviation. Initial optical density (600 nm) was 0.08 ±0.02 at 0 hr incubation. N=8 replicates per group.

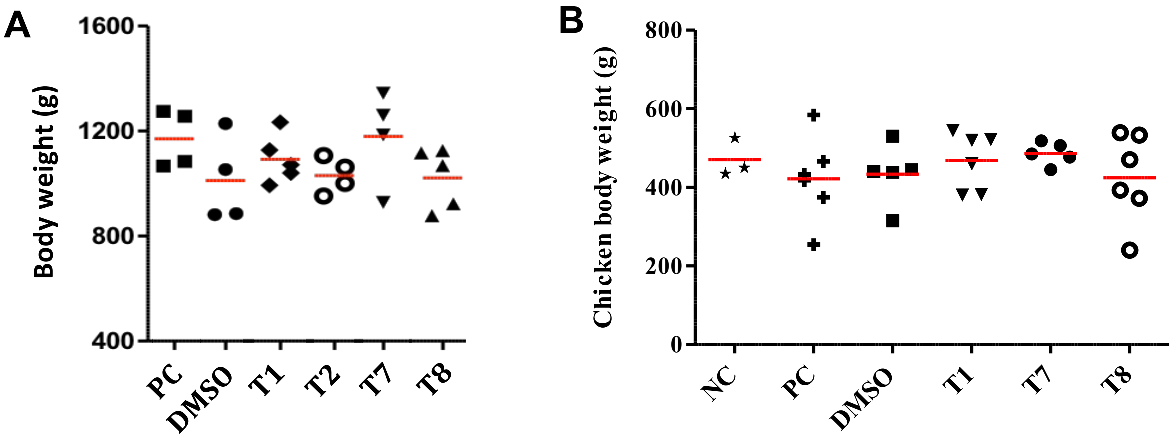

Supplemental Figure 2. A) Effect of the four most potent Tat-dependent inhibitors on five-week-old chicken body weight. B) Effect of the four most potent Tat-dependent inhibitors on three-week-old chicken body weight.

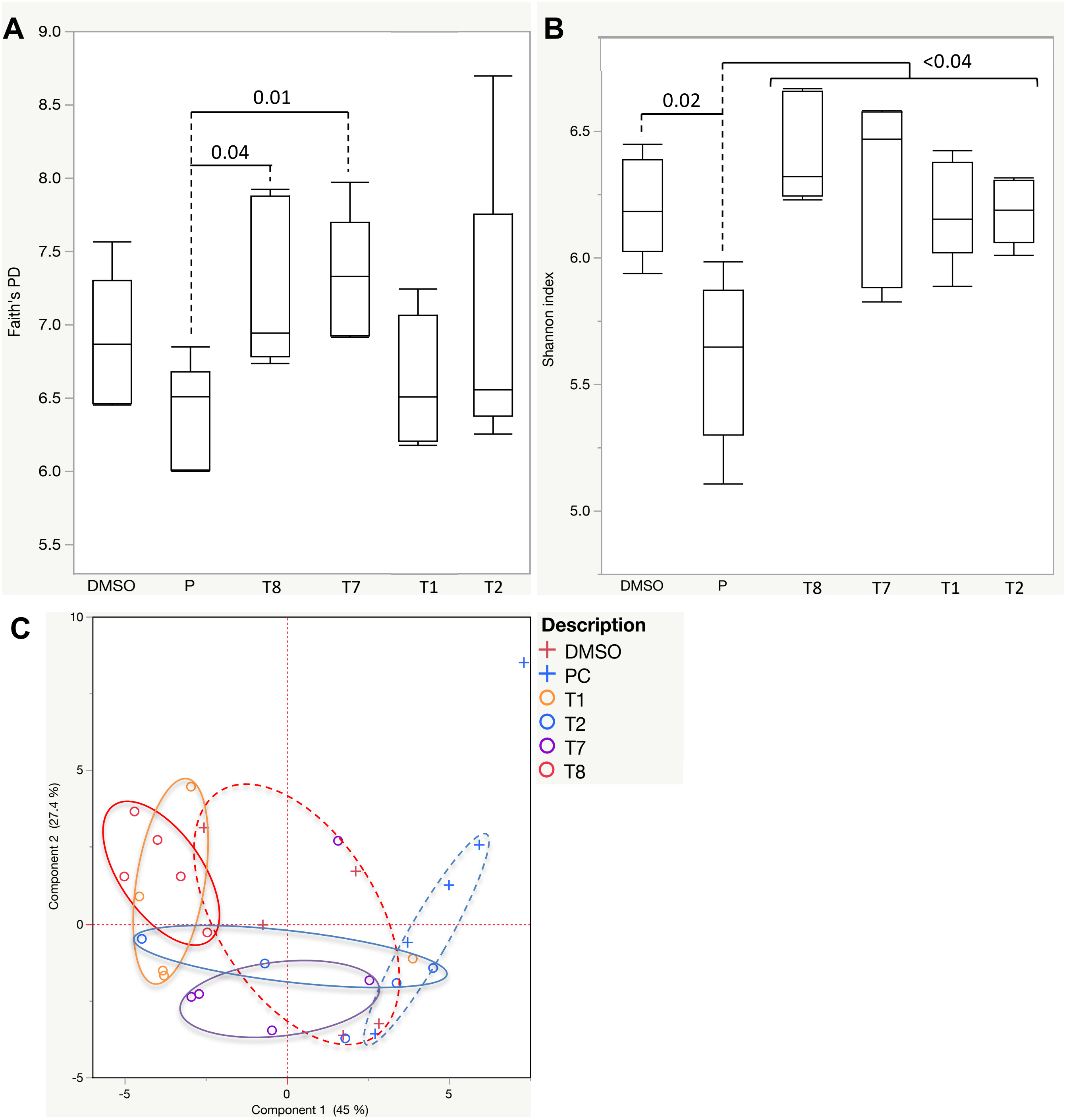
A
9.0
8.5
8.0
7.5
7.0
6.5
6.0
5.5
Faith's PD
0.01
0.04
DMSO
P
T8
T7
T1
T2
B
Shannon index
6.5
6.0
5.5
5.0
0.02
<0.04
DMSO
P
T8
T7
T1
T2
C
10
5
0
-5
Component 2 (27.4 %)
-5
0
5
Component 1 (45 %)
Description
DMSO
PC
T1
T2
T7
T8

Supplemental Figure 3 A-C. Effect of the four most potent Tat-dependent inhibitors on the alpha and beta diversity of five-week-old chicken cecal microbiota. Cecal microbiota were collected from five-week-old chicken treated for five days of treatment with 0.255 mg/ml of SM, respectively. B) Faith's PD (phylodiversity) and C) Shannon's (richness) index diversity of five-week-old chicken cecal microbiota. D) Two-dimensional plot of the principal coordinate analysis of five-week-old chicken cecal microbiota using weighted uniFrac values. Each dot represent the microbiota of one chicken. NC: not *C. jejuni* colonized not treated chickens; PC: *C. jejuni* colonized not treated chickens; DMSO: *C. jejuni* colonized chickens treated with DMSO (approx. 0.0001% per chicken); T1, T2, T7, T8: *C. jejuni* colonized chickens treated with one of the selected SM resuspended in DMSO. In the five-week-old chicken experiment, the DMSO treatment (DMSO, T1 and T7 groups) significantly decreased the abundance of *Lactobacillus* (between 16%-29%) compared the to the non-infected group (NC; $P<0.01$). T8 was not included for microbiota study since it did not have positive impact on *C. jejuni* load in cecum.

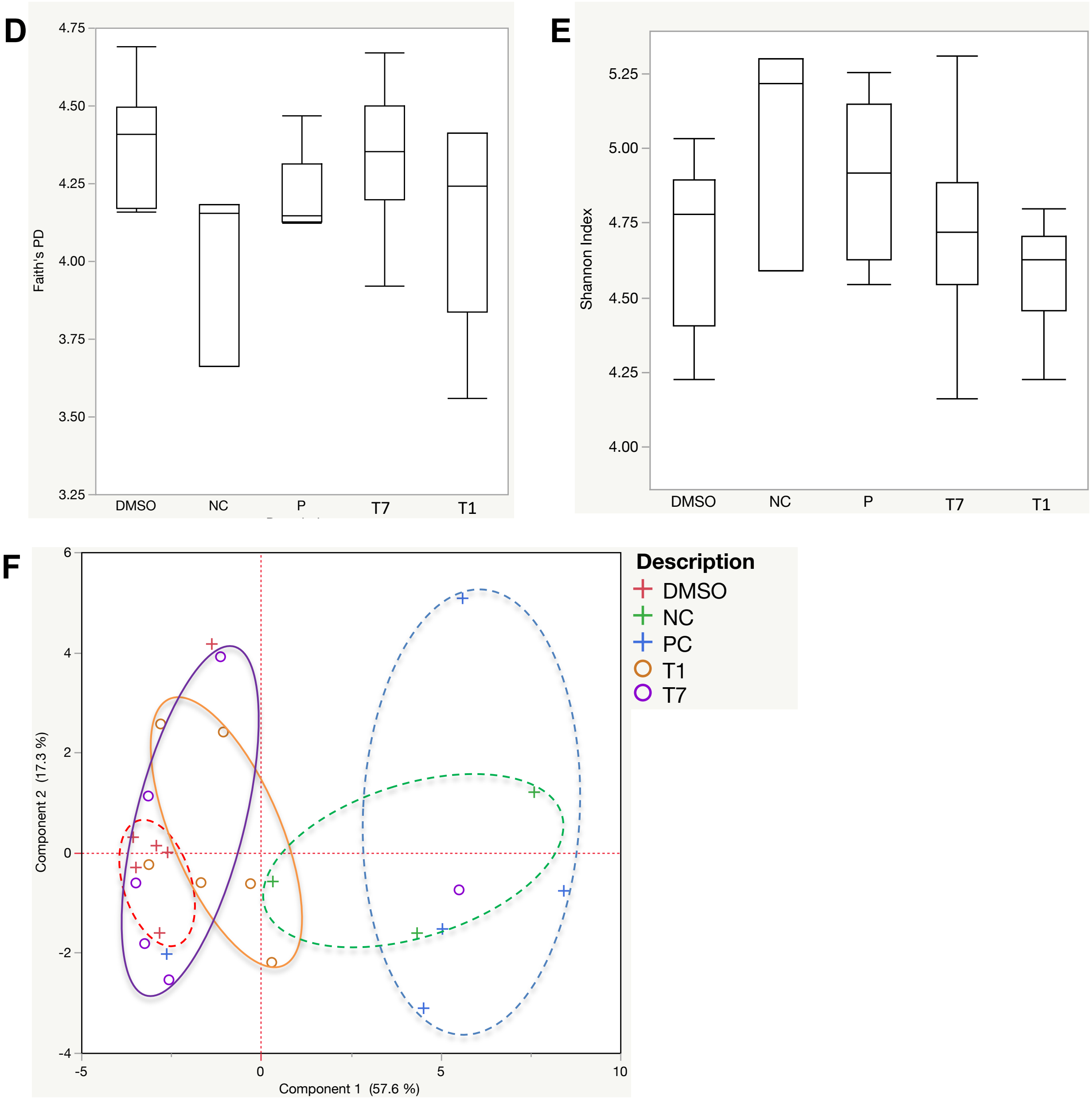

D
Faith's PD
4.75
4.50
4.25
4.00
3.75
3.50
3.25
DMSO
NC
P
T7
T1
E
Shannon Index
5.25
5.00
4.75
4.50
4.25
4.00
DMSO
NC
P
T7
T1
F
Component 2 (17.3 %)
6
4
2
0
-2
-4
-5
0
5
10
Component 1 (57.6 %)
Description
DMSO
NC
PC
T1
T7

Supplemental Figure 3 D-F continue. Effect of the four most potent Tat-dependent inhibitors on the alpha and beta diversity of three-week-old chicken cecal microbiota. Cecal microbiota were collected from three-week-old chicken treated for five days of treatment with 0.127 mg/ml of SM, respectively. D) Faith's PD (phylodiversity) and E) Shannon's (richness) index diversity of three-week-old chicken cecal microbiota. F) Two-dimensional plot of the principal coordinate analysis of three-week-old chicken cecal microbiota using weighted uniFrac values. Each dot represent the microbiota of one chicken. NC: not *C. jejuni* colonized not treated chickens; PC: *C. jejuni* colonized not treated chickens; DMSO: *C. jejuni* colonized chickens treated with DMSO (approx. 0.0001% per chicken); T1 and T2: *C. jejuni* colonized chickens treated with one of the selected SM resuspended in DMSO. In the three-week-old chicken experiment, the DMSO treatment (DMSO, T1 and T7 groups) had significant effect on the microbiome composition compared the chickens that were not treated with DMSO (NC and PC groups). More precisely, the abundance of Proteobacteria (between 12-19% increase), especially *Enterobacteriaceae* was significant higher in the DMSO treated groups (DMSO, T1 and T7) compared to the non-DMSO treated groups (NC and PC; P<0.01); while the opposite trend was observed with the Firmicutes, especially *Romboutsia* (between 5.5-6% decrease). In addition, groups infected with *Campylobacter* (PC, DMSO, T1 and T7) were characterized by significantly lower level in Firmicutes, especially *Clostridia* (between 11-22% reduction), [*Ruminococcus*] torques group (between 4.2-7% reduction), *Clostridium* sensu stricto 1 (between 7.3-13.2% reduction), *Ruminiclostridium* (between 1.3-2% reduction) and *Subdoligranulum* (between 2.9-4% reduction) compared to the non *C. jejuni* colonized group (NC; P<0.01).

Table S1: Strains used in these studies.

Strains	Relevant description	Source/Reference
C. jejuni 81-176 *wild-type*	MH broth; 42°C; micro-aerobic; 24 hrs	Laboratory collection
C. jejuni Δ*tatC* mutant	*C. jejuni* 81-176 *tatC* deletion mutant with kanamycin resistance marker	Rajashekara et *al.* 2009
Campylobacter jejuni Au-13, Au-20, Au-38, Au-39, Au-47, Au-50, Au-24, Au-32, Au-44 and Au-45	MH broth, 42°C, micro-aerobic for 24 hrs	[a]Dr. Patrick J. Backall
Campylobacter coli ATCC33559	MH broth, 42°C, micro-aerobic for 24 hrs	ATCC (pig feces)
Bifidobacterium lactis Bb12	MRS + Cysteine broth, 37° C, anaerobic for 24 hrs	[a]Christian Hansen Ltd.
Bifidobacterium adolescentis and *longum*	MRS + Cysteine broth; 37°C; anaerobic; 24 hrs	[b]Dr. Davis Francis
Enterococcus faecalis	MRS broth, 37° C, anaerobic for 18 hrs	[b]Dr. Davis Francis
Escherichia coli Nissle 1917	LB broth, 37° C, aerobic for 12 hrs	[c]Dr. Ulrich Sonnenborn
Levilactobacillus brevis	MRS broth; 37°C; anaerobic; 1- 2 days	[b]Dr. Davis Francis
Lacticaseibacillus rhamnosus GG	MRS broth; 37°C; anaerobic; 2 days	[e]ATCC

[a]Hørsholm, Denmark; [b]South Dakota State University, SD, USA; [c]Department of Biological Research, Ardeypharm GmbH, Germany; [d]Department of Microbiology, The Ohio State University; [e]American Type Culture Collection, Rockville, MD; MRS: Man Rogosa Sharpes; BHI: Brain Heart Infusion; LB: Luria Bertani; MH: Mueller-Hilton.

Rajashekara, G., Drozd, M., Gangaiah, D., Jeon, B., Liu, Z., and Zhang, Q. (2009). Functional Characterization of the Twin-Arginine Translocation System in Campylobacter jejuni. *Foodborne Pathog. Dis.* 6, 935–945. doi:10.1089/fpd.2009.0298.

Table S2. Libraries used for the identification of SM increasing the sensitivity of *C. jejuni* 81-176 to 0.5 mM copper sulfate .

Library	SM screened	SM concentrations tested (µg/ml)	% Hits (number of hits)
Asinex	12,378	6.25	1.95 (242)
Biomol 4	640	2.5	4.06 (26)
Chembridge 3	10,560	6.25	1.03 (109)
Chemdiv 4	14,677	6.25	0.80 (118)
Enamine 2 (partial)	3,520	6.25	1.05 (37)
LifeChemicals 1	3,893	6.25	0.98 (38)
MSDiscovery 1	270	6.25	2.96 (8)
Maybridge 5	3,212	6.25	1.37 (44)
Microsource 1	1,040	2.5	3.08 (32)
NIH Clinical Collection 1	446	12.5	2.24 (10)
NIH Clinical Collection 2	281	12.5	5.34 (15)

Hit: SM increasing the sensitivity of *C. jejuni* 81-176 to 0.5 mM copper sulfate.

Increased sensitivity: completely inhibited the growth of *C. jejuni* 81-176 only in presence of 0.5 mM copper sulfate.

Table S3. Details concerning the chicken groups used in three- and five-week-old chicken pilot studies.

**Treatments**	**Label**	**Doses**	**Age (days)[a]**	**Sample size**	***C. jejuni* inoculation[b]**	**Treatment[c]**
T1	T1	0.127 mg/kg	21	6	Yes	Yes
T7	T7	0.127 mg/kg	21	5	Yes	Yes
T8	T8	0.127 mg/kg	21	6	Yes	Yes
DMSO + *C. jejuni* colonized	DMSO	0.0001%	21	5	Yes	Yes
No DMSO + *C. jejuni* colonized	PC	None	21	6	Yes	No treatment
Non *C. jejuni* colonized non treated	NC	None	21	3	No	No treatment
T1	T1	0.225 mg/kg	39	5	Yes	Yes
T2	T2	0.225 mg/kg	39	4	Yes	Yes
T7	T7	0.225 mg/kg	39	5	Yes	Yes
T8	T8	0.225 mg/kg	39	5	Yes	Yes
DMSO + *C. jejuni* colonized	DMSO	0.0001%	39	4	Yes	Yes
No DMSO + *C. jejuni* colonized	PC	None	39	4	Yes	No treatment

[a] birds age (days) at the time of the inoculation; **[b]** Inoculum: 1 X 10^5 CFU of each *Campylobacter jejuni* strain per chicken (Au-12, Au-13, Au18, Au-21, and Au-35; Supplementary Table 1; [c] Treated orally twice a day for 5 days.

Table S4. Minimum inhibitory concentrations (MIC) of eight Tat dependent inhibitors against diverse *Campylobacter* strains in presence of 0.5 mM $CuSO_4$.

TAT SM	*Campylobacter* isolates											
	C. jejuni	***C. coli***	**Au-13**	**Au-20**	**Au-38**	**Au-39**	**Au-47**	**Au-50**	**Au-24**	**Au-32**	**Au-44**	**Au-45**
T1	0.19	0.31	1.25	0.31	0.31	0.31	0.16	0.31	0.16	0.62	0.31	0.31
T2	0.01	0.62	0.31	0.62	0.62	0.31	0.31	0.31	0.31	0.62	0.31	0.62
T3	0.04	1	6.25	6.25	6.25	6.25	6.25	6.25	6.25	6.25	6.25	6.25
T4	0.01	6.25	0.62	0.62	6.25	6.25	0.62	0.31	6.25	6.25	6.25	6.25
T5	0.01	0.02	0.62	2.5	2.5	5	2.5	5	2.5	5	2.5	5
T6	1.25	1.25	1.25	1.25	1.25	1.25	1.25	1.25	1.25	1.25	1.25	1.25
T7	0.02	0.16	0.31	0.16	0.16	0.31	0.31	0.08	0.08	0.08	0.31	0.16
T8	0.08	0.25	0.06	0.25	0.06	0.25	0.06	0.25	0.06	0.25	0.06	0.25

The values are expressed in μg/ml.

Table S5. Minimum bactericidal concentrations (MBC) of eight Tat dependent inhibitors against diverse *Campylobacter* strains in presence of 0.5 mM $CuSO_4$.

TAT SM	*Campylobacter* isolates											
	C. jejuni	***C. coli***	**Au-13**	**Au-20**	**Au-38**	**Au-39**	**Au-47**	**Au-50**	**Au-24**	**Au-32**	**Au-44**	**Au-45**
T1	0.62	0.62	2.5	0.62	0.62	0.62	0.32	0.62	0.32	1.24	0.62	0.62
T2	0.62	1.24	0.62	1.24	1.24	0.62	0.62	0.62	0.62	1.24	0.62	1.24
T3	>5	>5	>5	>5	>5	>5	>5	>5	>5	>5	>5	>5
T4	>5	>5	>5	>5	>5	>5	>5	>5	>5	>5	>5	>5
T5	0.04	0.04	1.24	5	5	>5	5	>5	5	>5	5	>5
T6	2.5	2.5	2.5	2.5	2.5	2.5	2.5	2.5	2.5	2.5	2.5	2.5
T7	0.16	0.32	0.62	0.32	0.32	0.62	0.62	0.32	0.16	0.16	1.24	0.32
T8	0.12	0.25	0.06	0.25	0.06	0.25	0.06	0.5	0.06	0.25	0.12	0.25

The values are expressed as µg/ml. >5.0: no bactericidal effect observed at 5 µg of compounds or lower in presence of 0.5 mM $CuSO_4$.
